# Postnatal Brain Trajectories and Maternal Intelligence Predict Childhood Outcomes in Complex CHD

**DOI:** 10.3390/jcm13102922

**Published:** 2024-05-15

**Authors:** Vincent K. Lee, Rafael Ceschin, William T. Reynolds, Benjamin Meyers, Julia Wallace, Douglas Landsittel, Heather M. Joseph, Daryaneh Badaly, J. William Gaynor, Daniel Licht, Nathaniel H. Greene, Ken M. Brady, Jill V. Hunter, Zili D. Chu, Elisabeth A. Wilde, R. Blaine Easley, Dean Andropoulos, Ashok Panigrahy

**Affiliations:** 1 Department of Bioengineering, University of Pittsburgh, Pittsburgh, PA 15260, USA; vkl2@pitt.edu; 2 Department of Radiology, University of Pittsburgh School of Medicine, Pittsburgh, PA 15260, USA; rcc10@pitt.edu (R.C.); william.reynolds@chp.edu (W.T.R.); benjamin.meyers2@chp.edu (B.M.); julia.wallace@chp.edu (J.W.); 3 Department of Biomedical Informatics, University of Pittsburgh School of Medicine, Pittsburgh, PA 15206, USA; 4 Department of Biostatistics, School of Public Health and Health Professions, State University of New York at Buffalo, Buffalo, NY 14260, USA; dplansit@buffalo.edu; 5 Department of Psychiatry, University of Pittsburgh, Pittsburgh, PA 15260, USA; liebherrh@upmc.edu; 6 Learning and Development Center, Child Mind Institute, New York, NY 10022, USA; daryaneh.badaly@childmind.org; 7 Division of Cardiothoracic Surgery, Children’s Hospital of Philadelphia, Philadelphia, PA 19104, USA; gaynor@chop.edu; 8 Perinatal Pediatrics Institute, Children’s National Hospital, Washinton, DC 20010, USA; dlicht@childrensnational.org; 9 Anesthesiology, Oregon Health and Science University, Portland, OR 97239, USA; nathanielgreene@me.com; 10 Department of Pediatrics and Department of Anesthesiology, Lurie Children’s Hospital, Northwestern University, Chicago, IL 60611, USA; brady.ken@gmail.com; 11 Department of Radiology, Baylor College of Medicine, Houston, TX 77030, USA; jvhunter@texaschildres.org (J.V.H.); zdchu@texaschildrens.org (Z.D.C.); elisabeth.wilde@hsc.utah.edu (E.A.W.); 12 H. Ben Taub Department of Physical Medicine and Rehabilitation, Baylor College of Medicine, Houston, TX 77030, USA; 13 Department of Neurology, University of Utah School of Medicine, Salt Lake City, UT 84132, USA; 14 Department of Pediatric Anesthesiology, Baylor College of Medicine, Houston, TX 77030, USA; beasley@texaschildrens.org (R.B.E.); dbandrop@texaschildrens.org (D.A.); 15 Department of Anesthesiology, Perioperative and Pain Medicine, Texas Children’s Hospital, Houston, TX 77030, USA

**Keywords:** congenital heart disease (CHD), structural brain trajectories, neurodevelopmental deficits, MRI, maternal IQ, white matter tract, regional brain volumes, diffusion tensor imaging

## Abstract

**Highlights:**

*Question:*

Do early infant brain trajectories in congenital heart disease (CHD) patients predict early childhood neurodevelopmental (ND) outcomes adjusted for known genetic abnormalities and maternal intelligence (IQ)?

*Findings:*

Reduced brain volumetric trajectories in infants with CHD predicted language outcomes at 5 years, adjusting for maternal IQ and genetic abnormalities. Maternal IQ substantially contributed to ND variance, nearly doubling from 1 year to 5 years.

*Meaning:*

Postnatal brain trajectories can predict early childhood ND in complex CHD. The influence of maternal IQ is cumulative and can exceed the influence of medical and genetic factors in CHD, underscoring the importance of not only heritable factors but also parent-based environmental factors.

**Abstract:**

**Objective:** To determine whether early structural brain trajectories predict early childhood neurodevelopmental deficits in complex CHD patients and to assess relative cumulative risk profiles of clinical, genetic, and demographic risk factors across early development. **Study Design**: Term neonates with complex CHDs were recruited at Texas Children’s Hospital from 2005–2011. Ninety-five participants underwent three structural MRI scans and three neurodevelopmental assessments. Brain region volumes and white matter tract fractional anisotropy and radial diffusivity were used to calculate trajectories: perioperative, postsurgical, and overall. Gross cognitive, language, and visuo-motor outcomes were assessed with the Bayley Scales of Infant and Toddler Development and with the Wechsler Preschool and Primary Scale of Intelligence and Beery–Buktenica Developmental Test of Visual–Motor Integration. Multi-variable models incorporated risk factors. **Results:** Reduced overall period volumetric trajectories predicted poor language outcomes: brainstem ((β, 95% CI) 0.0977, 0.0382–0.1571; *p* = 0.0022) and white matter (0.0023, 0.0001–0.0046; *p* = 0.0397) at 5 years; brainstem (0.0711, 0.0157–0.1265; *p* = 0.0134) and deep grey matter (0.0085, 0.0011–0.0160; *p* = 0.0258) at 3 years. Maternal IQ was the strongest contributor to language variance, increasing from 37% at 1 year, 62% at 3 years, and 81% at 5 years. Genetic abnormality’s contribution to variance decreased from 41% at 1 year to 25% at 3 years and was insignificant at 5 years. **Conclusion:** Reduced postnatal subcortical–cerebral white matter trajectories predicted poor early childhood neurodevelopmental outcomes, despite high contribution of maternal IQ. Maternal IQ was cumulative over time, exceeding the influence of known cardiac and genetic factors in complex CHD, underscoring the importance of heritable and parent-based environmental factors.

## 1. Introduction

Congenital heart disease (CHD) poses lifelong risks for complications, including neurodevelopmental deficits [1,2]. Children with CHD experience heterogenous degrees of neurodevelopmental outcomes, with language impairment the most prominent during early childhood [3,4]. Given the persistent risk of neurodevelopmental deficits in CHD, it is imperative to evaluate long-term developmental trajectories to understand and potentially intervene in relation to quality of life.

In individuals with CHD, alterations to the trajectory of brain development begin in utero, evolve over time, and are compounded by additional injury. These interact with a multitude of medical and demographic factors and lead to a heterogeneous array of adverse neurodevelopmental outcomes. Improved understanding of brain trajectories in the context of these factors provides us with a conceptual framework for the development of targeted interventions that might improve outcomes. Cross-sectional studies of CHD patients in infancy have shown that delayed brain maturity [5,6,7,8], reduced volumes [9,10,11], and abnormality in connectivity [12,13,14] are clearly associated with adverse neurodevelopment in CHD [15]. There are also a few studies examining brain growth trajectories between late fetal and neonatal periods [16,17,18], preoperative and postoperative time points [19], and neonatal to infant periods showing some indication of reduced brain volume trajectory [11]. However, there needs to be more trajectory studies assessing the predictive abilities of neonatal brain imaging in CHD for short- and long-term neurodevelopmental outcomes.

Past research, which has included extensive studies of multiple sources of exposures, has failed to adequately predict developmental delays, with significant unexplained (>60%) [2,4] variance in developmental outcomes. It is well established that social–environmental factors such as parenting, maternal education, and socioeconomic status are strong contributors to infant and child development, and there is emerging evidence to suggest the effect is even greater in those infants and children with CHD [20,21,22,23]. Maternal intellectual ability, which has been poorly understood in CHD cohorts, also has direct influence on children’s intellectual development because it is a genetically based and heritable trait [24]. Intellectual ability is also associated with adult life-course outcomes [25] that, in turn, shape the child-rearing environment [26].

Here, we primarily tested the hypotheses that reduced trajectory of early infant brain structures, as measured with MRI, would be predictive of early childhood neurocognitive outcomes at five years of age, adjusting for maternal IQ and genetic abnormalities (microdeletions) and other medical factors that are known to contribute to neurodevelopmental outcome variance. We secondarily tested the hypotheses that postnatal brain trajectories and concomitant influences of demographic factors on early neurodevelopmental outcomes are cumulative (i.e., the strength of effect increases with increasing age) and could potentially exceed the influence of medical and genetic factors in CHD. As such, we also correlated the same brain trajectories with comparable late infant (1 year) and toddler (3 years) neurodevelopmental outcomes within the same cohort, adjusting for similar covariates. Lastly, we also examined the predictive value of more perioperative and postsurgical brain trajectories with comparable sequential neurodevelopmental domains adjusting for similar covariates.

## 2. Methods

The study protocol, including all clinical data collection, acquisition of MRI data under sedation, and neurodevelopmental outcome testing of subjects and maternal IQ testing, was approved by the Baylor College of Medicine Institutional Review Board, and written informed parental consent was obtained. Written informed consent was obtained from a parent under Baylor College of Medicine IRB Protocols H-18531 (approval date 26 May 2006) and H-17115 (approval date 6 July 2005).

### 2.1. Study Population

Neonates diagnosed with hypoplastic left heart syndrome (HLHS—requiring Norwood operation and Blalock–Taussig shunt placement) or transposition of the great arteries (TGA—requiring arterial switch operation or ventricular septal defect repair with aortic arch reconstruction) were recruited into this study within the first 30 days of life at Texas Children’s Hospital from November 2005 to December 2011. Exclusion criteria were: (1) gestational age less than 35 weeks, (2) weight less than 2.0 kg at birth, (3) recognizable dysmorphic syndrome, and (4) preoperative cardiac arrest greater than 3 min.

### 2.2. Magnetic Resonance Imaging Studies

The study design included three MRIs performed under anesthesia: (1) the preoperative scan acquired just prior to surgery, after tracheal intubation (39.42 ± 1.53 weeks postconceptual age at scan); (2) the postoperative #1 scan from 7–10 days postoperatively (40.66 ± 1.53 weeks) under pentobarbital sedation; and (3) postoperative #2 scan from 4–6 months of life (61.34 ± 7.51 weeks) under pentobarbital or propofol infusions. All MRI scans were acquired on the same 1.5T Intera scanner (Philips Medical Systems, Best, Netherlands). Each of the scans included volumetric 3-dimensional T1-weighted and diffusion imaging (15 gradient directions B = 860 s/mm). Detailed MRI sequence parameters are presented as Supplemental Material (Table S1). All T1-weighted and diffusion images underwent in-house semi-automated segmentation or tractography pipelines as previously reported [6,27] (See Supplemental Methods for more details, Figures S1 and S2). The following brain regions were included: cerebral cortex, cerebral white matter volume (WMV), brainstem, cerebellum, intracranial cerebrospinal fluid (CSF), deep grey matter (DGM), and whole brain with and without CSF. The following white matter tracts were included: genu, body, and splenium of the corpus callosum (CC); left and right cortico-spinal tract (CST-L and CST-R); left and right fronto-occipital fasciculus (FOF-L and FOF-R); left and right inferior longitudinal fasciculus (ILF-L and ILF-R); and left and right superior longitudinal fasciculus (SLF-L and SLF-R). The average fractional anisotropy (FA) and radial diffusivity (RD) values for each white matter tract were calculated.

### 2.3. Trajectory of Structural Imaging Metrics

The trajectory of change in volume, FA, and RD were defined as change in imaging measurement (FA-index and mm^2^/s, respectively) over change in time between the two scans that encompass the trajectory period. The primary trajectory exposure for this study was the early infant trajectory period which was calculated spanning between preoperative and postoperative #2 scans. The secondary exposures were postsurgical trajectory (period between postoperative #1 and postoperative #2 scans) and perioperative trajectory (between preoperative and postoperative #1 scans). Because of the known dramatic differential increase in normative metrics in regional brain volume and DTI metrics during this period, no attempt was made to model all three time points into one trajectory measure.

### 2.4. Neurodevelopmental Tests

The participants underwent neurodevelopmental assessment at one, three, and five years of age. At one and three years, the Bayley Scales of Infant and Toddler Development, 3rd Edition (Bayley-III) was administered for indices of global cognitive, language, and motor functions. At 5 years, children completed the Wechsler Preschool and Primary Scale of Intelligence, 3rd edition (WPPSI-III) and Beery–Buktenica Developmental Test of Visual–Motor Integration, 6th edition (Beery VMI). To facilitate a conceptual comparison with 1- and 3-year outcomes, we focused our analyses on the WPPSI-III Full Scale IQ (as an index of global cognition), the WPPSI-III Verbal IQ (as an index of language-based skills), and the Beery VMI (as a measure of visual and motor skills). For each of the neuropsychological tests, norm-referenced scores were computed, comparing children to same-age peers.

### 2.5. Non-Imaging Factors

The non-imaging factors considered were factors previously recognized to be associated with neurodevelopmental outcomes in CHD: presence or absence of genetic abnormalities in general, and 22q deletion specifically; heart lesion type as single (HLHS) or double (TGA) ventricle; surgical variables—length of hospital stay, whether the participant had open heart surgery; demographic factors—SES status (assessed with Hollingshead Four-Factor Index of Socioeconomic Status); maternal IQ (assessed with Wechsler Abbreviated Scale of Intelligence, Second Edition; WASI-II); sex; race; ethnicity; birth weight; and white matter injury (WMI).

### 2.6. Statistical Analysis

Statistical analysis was performed using SAS software, version 9.4 (SAS Institute Inc.). A priori, we determined that the most relevant comparison was between the early infant imaging trajectory, which spans the longest duration between scans, and 5-year outcome, which represents the farthest neurodevelopmental assessment in this study. To assess whether brain imaging trajectories—and other factors that might impact neurodevelopment—predict neurodevelopmental outcomes, a multi-variable model was developed. This model examined the contribution of each imaging trajectory to each neurodevelopmental outcome while incorporating a set of non-imaging risk factors likely to contribute to the neuropsychological assessment variability as covariates. The covariate selection involves further refinement of risk factors demonstrating significant associations with neurodevelopmental tests and is as follows. An initial univariable regression analysis was conducted between each non-imaging risk factor (listed in Section 2.5 above) and each neurodevelopmental test. Following this, risk factors that demonstrated significant associations (alpha < 0.05) with neurodevelopmental outcomes are processed through collinearity tests and screening variable elimination (See Supplemental Results for more details). Consequently, maternal IQ, genetic abnormality, and cardiac ventricles to model HLHS-TGA status were incorporated into the final model. Post hoc factor contribution analysis was conducted for each multi-variable regression test that demonstrated significant association between imaging trajectory and neuropsychological test (See Supplemental Methods for more details, Equation (S1)). This analysis examined the contributions of each independent variable—imaging as well as non-imaging in the final multi-variable model—to the variance in test performance.

## 3. Results

The study cohort was 95 (42 (44.2%) female) neonatal patients (mean gestational age at birth of 38.23 ± 1.23 weeks) with demographics provided in Table 1. Due to attrition in imaging and follow-up neurodevelopmental assessments, the participant numbers in our multi-variable models were reduced. Our primary multi-variable models comparing early infant trajectory imaging to neurodevelopmental outcomes had: 54 (23 (42.6%) female) patients at 1-year, 39 (15 (38.5%) female) at 3-year, and 36 (16 (44.4%) female) at 5-year assessments. Our secondary models comparing postsurgical trajectory imaging to neurodevelopmental outcomes had 50 (22 (44.0%) female) patients at 1-year, 37 (16 (43.2%) female) at 3-year, and 36 (16 (44.4%) female) at 5-year assessments. The secondary models comparing perioperative trajectory imaging to neurodevelopmental outcomes had 48 (20 (41.7%) female) patients at 1-year, 36 (13 (36.1%) female) at 3-year, and 36 (15 (41.7%) female) at 5-year assessments. Detailed cohort information and summary statistics are provided in Supplementary Materials (See Supplemental Results Figure S3, and Tables S2–S7. Detail information about co-variate selection are provided in Supplementary Materials (see Supplemental Results Tables S8–S15). WMI incidence and volumes were not predictive of neurodevelopmental outcomes at any time points (See Supplemental Results Tables S16–S19). There were attritions within the study cohort for neurodevelopmental testing in successive follow up periods, but comparison analysis showed no significant differences in imaging trajectories and sociodemographic differences between those who had neurodevelopmental testing and those without neurodevelopmental testing. (See Supplementary Results Tables S20–S27).

### 3.1. Early Infant Brain Trajectories (Overall Period) and Childhood Outcomes

**Figure 1:**
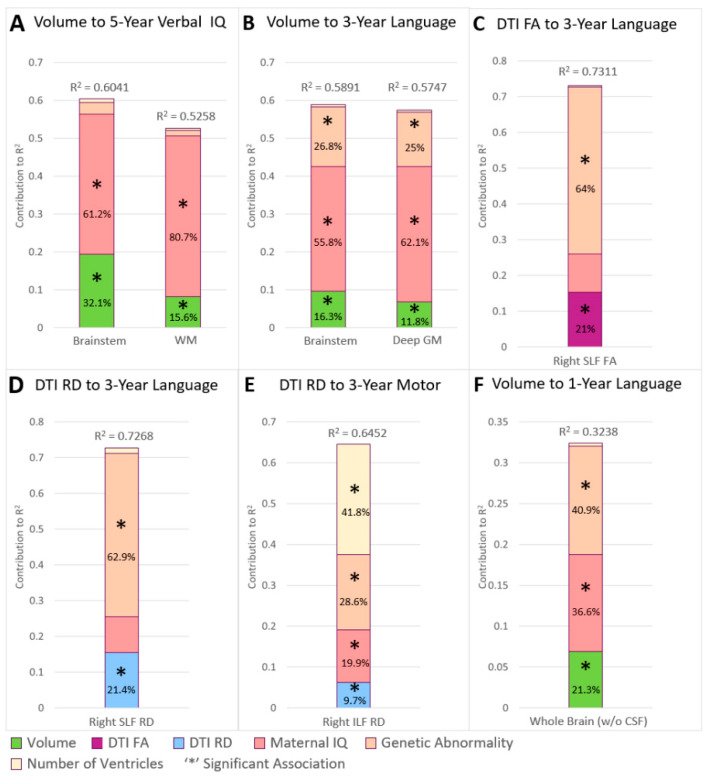
Early infant (overall period) multi-modal imaging trajectories and non-imaging factors’ contribution to variability in early childhood (**A**–**E**) and infant (**F**) outcomes. Reduced early infant (overall period) volume trajectories predicted poor language outcome across all three neurodevelopmental outcome periods (5-year, 3-year, and 1-year outcomes). Altered WM microstructural early infant (overall period) trajectories in SLF predicted poorer performance on a measure of language and ILF predicted poor motor performance at the 3-year assessment. Macrostructure (volume) trajectories in this period predicted 1-year (up to 21% of variability) and early childhood (up to 32% of variability) performance variability in language/verbal outcomes. The only significant contribution of cardiac lesion type (number of ventricles) was to 3-year motor outcomes. As such, early infant neuroimaging trajectory variables showed remarkable consistency in the degree of ND outcome variance explained across 1-year, 3-year, 5-year time points. Genetic abnormality’s contribution to outcome relatively decreased from 3-year to 5-year time points. In contrast, maternal IQ contributed to the outcomes, and the contribution to variability clearly showed a marked cumulative increase over time from the 1-year assessment (37%) to early childhood (up to 81%).

Reduced trajectory of brainstem (*p* = 0.0022; factor contribution (FC) = 32.1%; please see Table 2 for estimates and confidence intervals) and WM volume (*p* = 0.0397; FC = 15.6%) predicted poor 5-year language performance (Figure 1) in the final multi-variable model. Lower maternal IQ (*p* < 0.0001; FC up to 80.7%) was also predictive of poor 5-year language performance.

Reduced trajectory of brainstem (*p* = 0.0134; FC = 16.3%) and DGM (*p* = 0.0258; FC = 11.8%) volume predicted poor 3-year language performance in the final multi-variable model. In these analyses, lower maternal IQ (*p* < 0.0001; FC up to 62.1%) and presence of genetic abnormality (*p* = 0.002. FC up to 26.8%) also predicted poor language outcome. Additionally, reduced FA trajectory (*p* = 0.0256; FC = 21%) and increased RD trajectory (*p* = 0.0284; FC = 21.4%) of the SLF-R predicted poor 3-year language performance in the final multi-variable model. In these analyses, presence of genetic abnormality predicted poor language (*p* = 0.0011; FC up to 64%) performance as well. Besides language, increased RD trajectory (*p* = 0.0375; FC up to 9.7%) of the ILF-L predicted poor 3-year motor performance, which was also associated with lower maternal IQ (*p* = 0.0042; FC = 19.9%), presence of genetic abnormalities (*p* = 0.0009; FC = 28.6%), and single ventricle status (*p* = 0.0001; FC = 41.88%).

### 3.2. Early Infant Brain Trajectories (Overall Period) and Infant Outcomes

Reduced whole brain volume trajectory (*p* = 0.0286; FC = 21.3%) predicted poor 1-year language outcome (Table 2, Figure 1). Within this analysis, low maternal IQ (*p* = 0.0048; FC = 36.6%) and presence of genetic abnormality (*p* = 0.003; FC = 40.9%) co-contributed to the poorer 1-year language performance.

### 3.3. Postsurgical Brain Tractography and Childhood Outcomes

**Figure 2:**
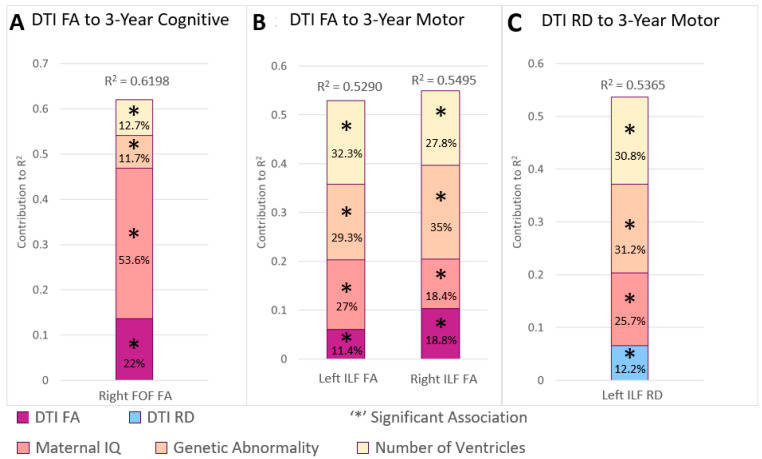
Postsurgical period imaging trajectories and non-imaging factors’ contribution to variability in early childhood outcomes. The postsurgical period imaging trajectories did not predict 5-year or 1-year outcomes. Volume trajectories from this period had no significant associations with any neurodevelopmental outcomes. Microstructure FA and RD accounted for up to 22% of cognitive and 19% of motor variability in 3-year outcomes. Maternal IQ contributed up to 54% in cognitive and 27% motor outcomes at 3 years. Genetic abnormality and number of cardiac ventricles were also co-contributors to 3-year outcome variability for this period.

In the postsurgical period imaging trajectories, the only significant findings were between tractography changes and 3-year early childhood cognitive and motor outcomes (Table 2, Figure 2). Reduced FA trajectory of the FOF-R (*p* = 0.0082; FC = 22%) predicted—along with lower maternal IQ (*p* = 0.0001; FC = 53.6%), presence of genetic abnormality (*p* = 0.0472; FC = 11.7%), and single ventricle status (*p* = 0.0379; FC = 12.7%)—poor 3-year cognitive outcome. Reduced FA trajectory of ILF-L (*p* = 0.0354; FC = 11.4%) and ILF-R (*p* = 0.0156; FC = 18.8%) and an increased RD trajectory of ILF-L (*p* = 0.0269; FC = 12.2%)—along with low maternal IQ (*p* = 0.0165, FC up to 27%), presence of genetic abnormality (*p* = 0.0015, FC up to 35%), and single ventricle status (*p* = 0.004, FC up to 32.3%)—predicted poor motor outcome (Figure 2B,C).

### 3.4. Perioperative Brain Trajectory and Childhood Outcomes

**Figure 3:**
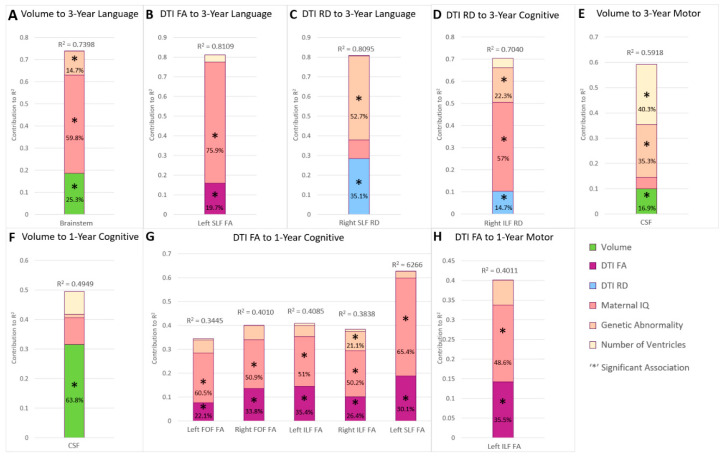
Perioperative period multi-modal imaging trajectories and non-imaging factors’ contribution to variability in early childhood (3-year) (**A**–**E**) and infant (1-year) (**F**–**H**) outcomes. The perioperative period imaging trajectories did not predict 5-year outcomes. Macrostructural trajectories (volume) contributed up to 64% in cognitive outcomes (CSF volume increase in (**F**)) at 1 year, as well as 17% of motor (CSF in (**E**)) and 25% of language variability (brainstem volume decrease in (**A**)) at 3 years. Microstructural FA trajectories accounted for up to 30% of cognitive (**G**) and 36% of motor (**H**) variability at 1 year and 20% of language (**B**) variability at 3 years. Microstructure RD trajectories contributed up to 15% of cognitive (**D**) and 35% of language (**C**) outcomes at 3 years. As such, perioperative neuroimaging trajectory variables showed remarkable consistency in degree of neurodevelopmental outcome variance explained across 1-year and 3-year time points, with the exception of increased CSF. Genetic abnormality’s contributions to cognitive outcome variability across the microstructure trajectory models were relatively consistent from 1-year to 3-year time points (21% to 22%), with the exception of language outcomes at 3 years. In contrast, maternal IQ’s contribution to outcome variability clearly showed a cumulative increase over time from 1 year to 3 years.

In the perioperative imaging trajectories, reduced trajectory of brainstem volume (*p* = 0.0111; FC = 25.3%) as well as reduced FA trajectory of SLF-L (*p* = 0.009, FC = 19.7%) and increased RD trajectory of SLF-R (*p* = 0.0296, FC = 35.1%), predicted poor 3-year language performance (Table 2, Figure 3). Along with the imaging findings within these models, low maternal IQ (*p* < 0.0001, FC up to 75.9%) and presence of genetic abnormality (*p* = 0.0125, FC up to 52.7%) also predicted poor 3-year language performance. Within this imaging epoch, increased RD trajectory of ILF-R (*p* = 0.0076; FC = 14.7%)—along with maternal IQ (*p* < 0.0001, FC = 57%) and presence of genetic abnormality (*p* = 0.0014, FC = 22.3%)—predicted poor 3-year cognitive outcome. Meanwhile, increased trajectory of CSF volume (*p* = 0.0365, FC = 16.9%)—along with presence of genetic abnormality (*p* = 0.0044, FC = 35.3%) and single ventricle status (*p* = 0.0044, FC = 40.3%)—predicted poor 3-year motor performance.

### 3.5. Perioperative Period Brain Trajectory and Infant Outcomes

Increased CSF volume (*p* = 0.0062, FC 63.8%) predicted poorer 1-year cognitive outcome (Table 2, Figure 3). Reduced FA trajectory of FOF-L (*p* = 0.0402, FC = 22.1%), FOF-R (*p* = 0.0089, FC = 33.8%), ILF-L (*p* = 0.0043, FC = 35.4%), ILF-R (*p* = 0.0112, FC = 26.4%), and SLF-L (*p* = 0.0042, FC = 30.1) predicted poor 1-year cognitive outcomes. Within these findings, lower maternal IQ (*p* = 0.0001, FC up to 65.4%)—and presence of genetic abnormality (*p* = 0.0222, FC = 21.1%) with only the ILF-R finding—also contributed to poor 1-year cognitive outcome.

## 4. Discussion

Prior studies have shown that neurodevelopmental impairments—manifesting as deficits in gross cognitive, visuo-motor, and language functions—are common in children with CHD in infancy and early childhood [15,28,29]. These studies also linked these neurodevelopmental impairments with brain dysmaturation—characterized by MRI biomarkers of qualitative assessments and quantitative volume and WM tractography measures—in the early neonatal period [6,30,31,32]. However, in these studies, the imaging biomarkers have been modeled as individual exposures on a single time point basis, even in studies that acquired multiple scans. We demonstrated modeling early infant brain trajectories can prognosticate childhood neurodevelopmental outcomes, even after adjusting for important patient-specific, known genetic, and socio-environmental factors. The early-infant trajectory encapsulates the microstructural and macrostructural changes the neonatal CHD brain experiences from just after birth to approximately six months of life, which is a major critical period of brain development. We show regions of higher vulnerability include not only white matter structures but also subcortical structures, which is consistent with prior infant CHD neuroimaging literature [33,34,35].

For non-imaging covariates, maternal IQ had the most significant contribution in the multi-variable model, followed by the presence of genetic abnormalities, while number of cardiac ventricles had the least significant predictions. Although maternal IQ has previously been collected in a CHD related study [36], our analysis is the first to model the quantitative effects of maternal IQ in this population. Parental SES (combined measure of marital and employment status, educational attainment, and occupation prestige) is often used as a proxy for environmental factors and has been shown to influence neurodevelopmental outcomes in other studies [37,38,39]. We found that parental SES was highly colinear with maternal IQ but had significantly fewer correlations with outcomes [40]. Prior research has found that “cognitively stimulating environments” [41] are associated with the development of intelligence, and while this is correlated with educational attainment and occupational prestige, additional factors such as education quality, parental expectations for academic achievement, and parental scaffolding may explain the unique contribution of maternal IQ above and beyond parent SES [40,42]. Furthermore, higher maternal IQ may be involved in greater parental involvement and further enrichment of a child’s cognitive stimulation. This early stimulation can positively impact CHD neurodevelopmental outcomes as recently demonstrated by a neuroimaging study [43]. Maternal IQ may also correlate with seeking better care for the child, or even earlier, in seeking prenatal care and making informed decisions during pregnancy. Adequate prenatal care is essential for healthy fetal development and can impact neurodevelopmental outcomes [44]. This protective and stimulating effect of maternal IQ is supported by our findings where maternal IQ was highly predictive of neurodevelopmental outcomes, and usually provided the highest contribution to the variance (R^2^). Furthermore, the contribution of maternal IQ to outcomes increased in proportion from one to five years, suggesting a cumulative effect on neurodevelopment over time. In contrast, the impact of genetic abnormalities diminishes during this same period. Genetic abnormality and single ventricle status are patient-specific conditions present in utero, and the fact that they are not predictive of neurodevelopmental outcomes beyond three years suggests that, by 5 years of age, the gross heritable mechanisms and environmental factors as indexed by maternal IQ might have had time to exert more significant influence on neurodevelopment and thereby counter the adverse effects of these patient factors.

We also found that modeling earlier brain trajectories identified critical periods of brain development during the perioperative and postsurgical periods, which predicted earlier ND outcomes (but not later 5-year outcomes). The perioperative period coincides with a period of neonatal neurogenesis that experiences rapid myelination, and perhaps because of this, microstructure trajectories dominate significant findings—with reduced cortical–cortical tract FA trajectories predicting poor ND outcomes at one year [7,8,14,45,46,47]. The postsurgical trajectory showed that reduced white matter microstructure predicted poor cognitive and motor outcomes at three years and may represent correlates of diffuse WMI as noted in preclinical surgical-based animal models of CHD [48,49]. In contrast, focal WMI, acquired in CHD infants on serial preoperative/immediate postoperative brain MRIs (usually performed on days 7–14 postnatally and are detected with 3D-T1-based MR imaging), involves punctate periventricular fronto-parietal white matter lesions [50,51,52,53,54,55,56]. Of note, in our study, focal WMI was not predictive of outcomes in this cohort, and this could be related to the overall incidence of these lesions decreasing over time, as recently described by Peyvandi et al. [57]. Additionally, the influence of positive sociodemographic factors could act as a potential cognitive reserve mechanism, limiting the impact of acquired brain injury patterns with neurocognitive outcomes, which has been noted by Latal et al. [58].

Our study has numerous strengths, including the prospective, longitudinal design, the solid and objective cognitive characterization of the patients and their parents, the racial and ethnic diversity of the sample, and the ability to perform serial scanning within a tight window. However, there are notable limitations. One limiting factor was the relatively high attrition rate, both in terms of lessening the sample size and the potential for introducing bias in neurodevelopmental testing. We have performed extensive secondary analyses showing that there are minimal baseline and early differences between the patients lost to follow-up and those who have been willing to return to follow-up. We also performed a comparison analysis to ensure no differences in imaging and non-imaging variables between those who were assessed and those who did not participate would skew or bias the results (see Supplementary Materials). Regardless, this attrition is likely to skew the analysis toward patients with higher maternal IQ and stronger cognition outcomes. For future work with a larger sample (potentially in collaboration with the Cardiac Neurodevelopmental Outcome Collaborative), we could run an analysis that would compare the trajectory of early neuroimaging and the trajectory of early neurodevelopmental testing to see which was more predictive of childhood neurodevelopmental outcomes relative to a cost analysis (given that MRI may be more expensive but is performed at a greater rate for clinical purposes).

## 5. Conclusions

We have shown that reduced trajectory of multi-modal neonatal brain maturation predicts poorer early childhood outcomes, especially language-based outcomes, despite the contribution of known genetic and demographic factors. Imaging trajectories, with their capability for earlier assessment than neuropsychological tests and the fact that these imaging trajectories can predict outcomes even up to 5 years, might be helpful as early biomarkers to predict neurodevelopmental outcomes to help guide intervention. Maternal IQ was cumulative over time, exceeding the influence of known innate cardiac and genetic factors in complex CHD, underscoring the importance of heritable and parental-based environmental factors.

## Figures and Tables

**Table 1:** Demographics and Risk Factors.

	Recruited Cohort (N = 95)	1-Year Cohort (N = 54)	3-Year Cohort (N = 39)	5-Year Cohort (N = 36)
Parental SES	40.66 ± 14.91	39.78 ± 14.52	41.89 ± 13.78	41.30 ± 13.63
Maternal IQ	102.92 ± 16.73	102.5 ± 17.01	104.90 ± 16.87	104.11 ± 18.17
Birth Weight (g)	3157 ± 489	3186 ± 513	3164 ± 462	3190 ± 487
Gestational Age at Birth (Weeks)	38.23 ± 1.23	38.04 ± 1.20	38.03 ± 1.25	38.19 ± 1.35
	Median (IQR)	Median (IQR)	Median (IQR)	Median (IQR)
Hospital Length of Stay (days)	26 (20–39)	24 (20–37)	23 (20–38)	23 (20–31)
Open Sternum Duration (days)	2 (1–2)	2 (1–2)	2 (1–2)	2 (1–2)
	Incident (%)	Incident (%)	Incident (%)	Incident (%)
Female Sex	42 (44.21%)	23 (0.43%)	15 (0.38%)	16 (0.44%)
Race and Ethnicity				
Ethnicity (Hispanic)	33 (35%)	16 (30%)	10 (26%)	10 (28%)
Race (White)	83 (87%)	48 (89%)	37 (95%)	34 (94%)
Race (Black)	10 (11%)	6 (11%)	2 (05%)	2 (06%)
Race (Asian)	2 (2%)	0 (0%)	0 (0%)	0 (0%)
Received Open Sternum Surgery	23 (24%)	10 (19%)	10 (26%)	8 (22%)
Any Genetic Abnormality *	19 (20%)	11 (20%)	10 (26%)	7 (19%)
22q Deletion	8 of 19 (8%)	5 of 11 (45%)	5 of 10 (50%)	4 of 7 (57%)
Cardiac Single Ventricles	49 (52%)	25 (46%)	19 (49%)	16 (44%)
White Matter Injury	41 (44%)	34 (63%)	27 (69%)	23 (64%)

* Other genetic abnormalities: 4q deletion, 19q deletion, 14q13.1 gain, xp22.31 gain, 8p23.1 gain, 7p21.3, 18q22.1 gain, 12q24.2 duplication, 45XO, 9pq21.3 gain, 9q. region duplication.

**Table 2:** Adjusted Regression Analysis of Multi-Modal Imaging Trajectories to Neurodevelopmental Outcomes.

Neurodevelopmental Outcomes	Imaging Biomarker	R^2^	Imaging Trajectory		Maternal IQ		Genetic Abnormality		Cardiac Ventricles	
			β Coefficient (95% CI)	*p*-Value	β Coefficient (95% CI)	*p*-Value	β Coefficient (95% CI)	*p*-Value	β Coefficient (95% CI)	*p*-Value
	**Early Infant Volume Trajectory**									
5 Year										
WPPSI-III Verbal IQ	Brainstem	0.6041	0.0977 (0.0382–0.1571)	0.0022	0.483 (0.27–0.696)	<0.0001	n.s.	0.1915	n.s.	0.4582
WPPSI-III Verbal IQ	WMV	0.5258	0.0023 (0.0001–0.0046)	0.0397	0.55 (0.32–0.78)	<0.0001	n.s.	0.3762	n.s.	0.5845
3 Year										
Bayley-III Language	Brainstem	0.5891	0.0711 (0.0157–0.1265)	0.0134	0.401 (0.232–0.57)	<0.0001	−10.9 (−17.53–−4.27)	0.002	n.s.	0.504
Bayley-III Language	DGM	0.5747	0.0085 (0.0011–0.016)	0.0258	0.45 (0.279–0.622)	<0.0001	−11.2 (−17.92–−4.48)	0.0018	n.s.	0.4888
1 Year										
Bayley-III Language	Whole Brain	0.3238	0.0008 (0.0001–0.0016)	0.0286	0.257 (0.082–0.432)	0.0048	−11.49 (−18.89–−4.1)	0.003	n.s.	0.5991
	**Postsurgical Volume Trajectory**—No Significant Findings									
	**Perioperative Volume Trajectory**									
3 Year										
Bayley-III Language	Brainstem	0.7398	0.0073 (0.0019–0.0126)	0.0111	0.419 (0.217–0.62)	0.0004	−6.05 (−11.92–−0.18)	0.044	n.s.	0.8218
Bayley-III Motor	CSF	0.5918	−0.0001 (−0.0003–0)	0.0365	n.s.	0.1495	−13.59 (−22.35–−4.84)	0.0044	11.78 (4.68–18.88)	0.0027
1 Year										
Bayley-III Cognitive	CSF	0.4949	−0.0002 (−0.0003–−0.0001)	0.0062	n.s.	0.1193	n.s.	0.5698	n.s.	0.1488
	**Early Infant DTI FA Trajectory**									
3 Year										
Bayley-III Language	SLF-R	0.7311	2263 (328–4199)	0.0256	n.s.	0.0556	−17.02 (−25.34–−8.69)	0.0008	n.s.	0.6985
	**Postsurgical DTI FA Trajectory**									
3 Year										
Bayley-III Cognitive	FOF-R	0.6198	3769 (1055–6483)	0.0082	0.351 (0.189–0.513)	0.0001	−6.44 (−12.79–−0.09)	0.0472	5.86 (0.3–11.43)	0.0397
Bayley-III Motor	ILF-L	0.529	2760 (201–5319)	0.0354	0.321 (0.127–0.514)	0.002	−13.75 (−21.71–−5.78)	0.0014	12.83 (5.75–19.92)	0.0009
Bayley-III Motor	ILF-R	0.5495	3178 (651–5705)	0.0156	0.255 (0.05–0.46)	0.0165	−13.46 (−21.31–−5.62)	0.0015	10.65 (3.7–17.6)	0.004
	**Perioperative DTI FA Trajectory**									
3 Year										
Bayley-III Language	SLF-L	0.8109	155 (46–263)	0.009	0.503 (0.324–0.683)	<0.0001	n.s.	0.9721	n.s.	0.166
1 Year										
Bayley-III Cognitive	FOF-L	0.3445	232 (11–452)	0.0402	0.396 (0.168–0.624)	0.0011	n.s.	0.0796	n.s.	0.576
Bayley-III Cognitive	FOF-R	0.401	331 (88–573)	0.0089	0.375 (0.151–0.599)	0.0017	n.s.	0.0738	n.s.	0.808
Bayley-III Cognitive	ILF-L	0.4085	326 (109–544)	0.0043	0.384 (0.171–0.597)	0.0008	n.s.	0.0933	n.s.	0.4537
Bayley-III Cognitive	ILF-R	0.3838	337 (81–593)	0.0112	0.424 (0.191–0.658)	0.0007	−10.92 (−20.19–−1.65)	0.0222	n.s.	0.4349
Bayley-III Cognitive	SLF-L	0.6266	257 (91–423)	0.0042	0.665 (0.374–0.957)	0.0001	n.s.	0.2241	n.s.	0.951
Bayley-III Motor	ILF-L	0.4012	340 (109–571)	0.005	0.389 (0.163–0.615)	0.0013	n.s.	0.0542	n.s.	0.8822
	**Early Infant DTI RD Trajectory**									
3 Year										
Bayley-III Motor	ILF-R	0.6452	−780 (−1511–−49)	0.0375	0.278 (0.096–0.46)	0.0042	−12.09 (−18.7–−5.48)	0.0009	12.97 (7.11–18.83)	0.0001
Bayley-III Language	SLF-R	0.7268	−697 (−1307–−87)	0.0284	n.s.	0.0695	−15.23 (−22.99–−7.47)	0.0011	n.s.	0.4574
	**Postsurgical DTI RD Trajectory**—No Significant Findings									
	**Perioperative DTI RD Trajectory**									
3 Year										
Bayley-III Cognitive	ILF-R	0.7040	−91 (−155–−27)	0.0076	−0.412 (0.264–0.560)	<0.0001	−9.64 (−15.18–−4.09)	0.0014	n.s.	0.0761
Bayley-III Language	SLF-R	0.8095	−66 (−125–−9)	*p*.0296	n.s.	0.1611	−12.37 (−21.13 –−3.61)	0.0125	n.s.	0.7573

Abbreviations: WMV—cerebral white matter volume; DGM—deep grey matter volume; FOF—fronto-occipital fasciculus; ILF—inferior longitudinal fasciculus; SLF—superior longitudinal fasciculus; L—left; R—right; FA—Fractional Anisotropy; RD—Radial Diffusivity; n.s.—not significant.

## Data Availability

The authors confirm that the data supporting the findings of this study are available within the article and its Supplementary Materials. Raw data were generated at the University of Pittsburgh. Derived deidentified data supporting the findings of this study are available from the corresponding author (V.L.) on request.

## Supplementary Materials

The following supporting information can be downloaded at: https://www.mdpi.com/article/10.3390/jcm13102922/s1, Figure S1: Diagram of the Neonatal Brain Structure Segmentation Pipeline; Figure S2: White Matter Tracts Generated by Tractography; Figure S3: Study Design and Cohort of Participants Imaging and Neurodevelopmental Testing Timeline; Equation (S1): Factor Contribution Analysis calculation; Table S1: MRI Sequence Parameters; Table S2: Brain Volumes Summary Statistics; Table S3: White Matter Tract DTI FA and RD Summary Statistics; Table S4: Neurodevelopmental Tests Summary Statistics; Table S5: Brain Volume Trajectories Summary Statistics; Table S6: White Matter Tract DTI FA Trajectories Summary Statistics; Table S7: White Matter Tract DTI RD Trajectories Summary Statistics; Table S8: Comparison of Neurodevelopmental Outcomes to Non-imaging Factors—Socio-Demographic; Table S9: Comparison of Neurodevelopmental Outcomes to Non-imaging Intrinsic Factors; Table S10: Comparison of Neurodevelopmental Outcomes to Non-imaging Perinatal Factors; Table S11: Comparison of Neurodevelopmental Outcomes to Medical Care & Surgery Factors; Table S12: Comparison of Neurodevelopmental Outcomes to Non-imaging Factors—White Matter Injury; Table S13: Correlation Among Non-Imaging Factors; Table S14: Maternal IQ Multi-variable Regression; Table S15: Parental SES Multivariable Regression; Table S16: Brain Volume Trajectory differences between the groups with and without WMI; Table S17: DTI FA Trajectory differences between the groups with and without WMI; Table S18: DTI RD Trajectory differences between the groups with and without WMI; Table S19: Neurodevelopmental Testing difference between those with WMI and those without WMI; Table S20: Early Infant Volume Trajectory Differences between Groups with and without Neurodevelopmental Testing; Table S21: Post-Surgical Period Volume Trajectory Differences between Groups with and without Neurodevelopmental Testing; Table S22: Perioperative Period Volume Trajectory Differences between Groups with and without Neurodevelopmental Testing; Table S23: Early Infant Period DTI Trajectory Differences between Groups with and without Neurodevelopmental Testing; Table S24: Post-Surgical Period DTI Trajectory Differences between Groups with and without Neurodevelopmental Testing; Table S25: Perioperative Period DTI Trajectory Differences between Groups with and without Neurodevelopmental Testing; Table S26: Sociodemographic Factor Differences between Groups with and without Neurodevelopmental Testing; Table S27: One-year and Three-year Neurodevelopmental differences between Participants that Attended Five-year Assessment and Those Who Did Not.
