# Supplementary material for: Postnatal Brain Trajectories and Maternal Intelligence Predict Childhood Outcomes in Complex CHD
Source: J Clin Med. 2024 May 15;13(10):2922. doi: 10.3390/jcm13102922 (PMC11121951; doi:10.3390/jcm13102922)
Supplement: Supplementary file 1 [file jcm-13-02922-s001.zip › jcm-2957802-supplementary.pdf]

Postnatal Brain Trajectories and Maternal Intelligence Predict Childhood Outcomes in Complex CHD

Supplementary Materials

Supplemental Methods

Table S1. MRI Sequence Parameters

Image Type	TR/TE (ms)	Flip angle (°)	FOV (mm)	Slice thickness/ gap (mm)	Number of slices	Acquisition/ reconstruction matrix size	Other
3D FFE T1-weighted sagittal	20/ 4.1	30	200	1.0/ 0.0	100	224/256	
15 direction axial DTI	10,071/90	90	256	2.7/ 0.0	55	96/128	b=0,860 (c/mm^2)

DTI – Diffusion tensor imaging; FFE – fast field echo; FOV – field of view; TE, echo time; TR, relaxation time;

Suppelemental Methods Regional Brain Volumes Segmentation

All T1's for each subject were processed through an in-house semi-automated segmentation pipeline, Neonatal Brain Structure Segmentation (NeBSS)[1] – an open-source Python project and is freely available at www.github.com/PIRCImagingTools/NeBSS. Additionally, a Docker image and Dockerfile are also available. Subject T1 images are first pre-processed using FSL's Brain Extraction Tool (BET)[2] and FSL FAST[3] for bias correction. After pre-processing, NeBSS has two branches, Branch A which outputs structural volumes using the ALBERT Brain Atlas[4], and Branch B which outputs probabilistic tissue volumes using the Probabilistic Neonatal Brain Atlas[5]. Within Branch A, four atlas images, chosen based on age similarity to the subject's gestational age, are transformed into subject space using Advanced Normalization Tools (ANTs) non-linear transformation algorithm[6]. A voxel-wise winner takes all approach is performed and a winner is chosen at random if there is a tie. The resulting segmentation has 50 non-overlapping regions. After completion, inspection and manual correction for the cerebellum, amygdala, and hippocampus is carried out. Branch B works in the opposite direction, mapping the subject into the probabilistic atlas space, again utilizing ANTs non-linear transformation algorithm. Threshold values for each tissue map are manually determined and binarized at that threshold value. Branch B outputs 10 volumes from independent probabilistic tissue maps. The schematic of this image processing pipeline is presented in Figure S1.

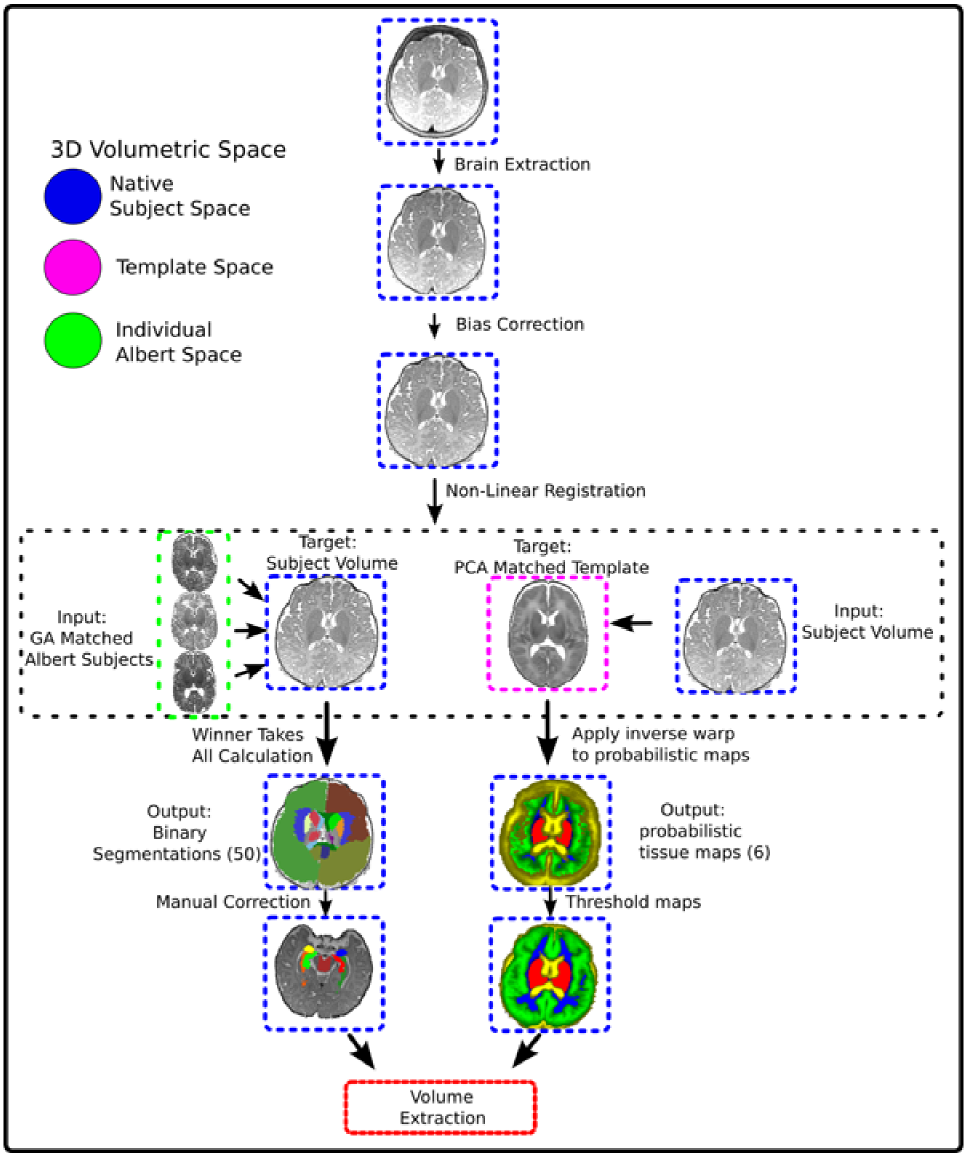

Figure S1. Diagram of the Neonatal Brain Structure Segmentation Pipeline.
Diagram showing the Neonatal Brain Structure Segmentation (NeBSS) pipeline used to generate the volume measurements of the various regions. The two parallel processes in the lower half of the diagram illustrates the Winner Takes All (left) and probabilistic maps (right) algorithms used to generate outputs that are ultimately combined to provide extracted volumes of interest.

Suppelemental Methods White Matter Tractography

Each DTI acquisition was manually inspected for artifacts, and affected gradient volumes were removed. Each was eddy current and motion corrected with DSI Studio[7] and reconstructed using the diffusion tensor algorithm. The processed DTI of each participant was used to generate a set of white matter tracks with an in-house automated tractography pipeline which was appropriate for the age-span of this study. A brief explanation of the pipeline is as follows. Fractional anisotropy (FA) maps were generated for each participant from the processed DTI. The participant whose FA map has the lowest deformation – after the process of registering each subject's FA map to every other subject using FSL[8] FLIRT[2] and FNIRT[9] – is chosen as the most representative subject. This representative subject's FA map is used as the template space to draw the dedicated sets of regions of interests (ROI) and regions of avoidance (ROA) – which are necessary for generation of individual white matter tracks. These sets of ROI and ROA are then inverse transformed, using ANTS[10], and back projected onto each participant's DTI space to generate subject specific set of white matter tracks. Deterministic tractography was conducted in DSI Studio with the following parameters: FA threshold = 0.1, angle threshold = 45°, step size = 1mm, without smoothing, max tracts = 5,000, max seeds = 10,000,000. Example tractography images for these tracts are presented in Figure S2.

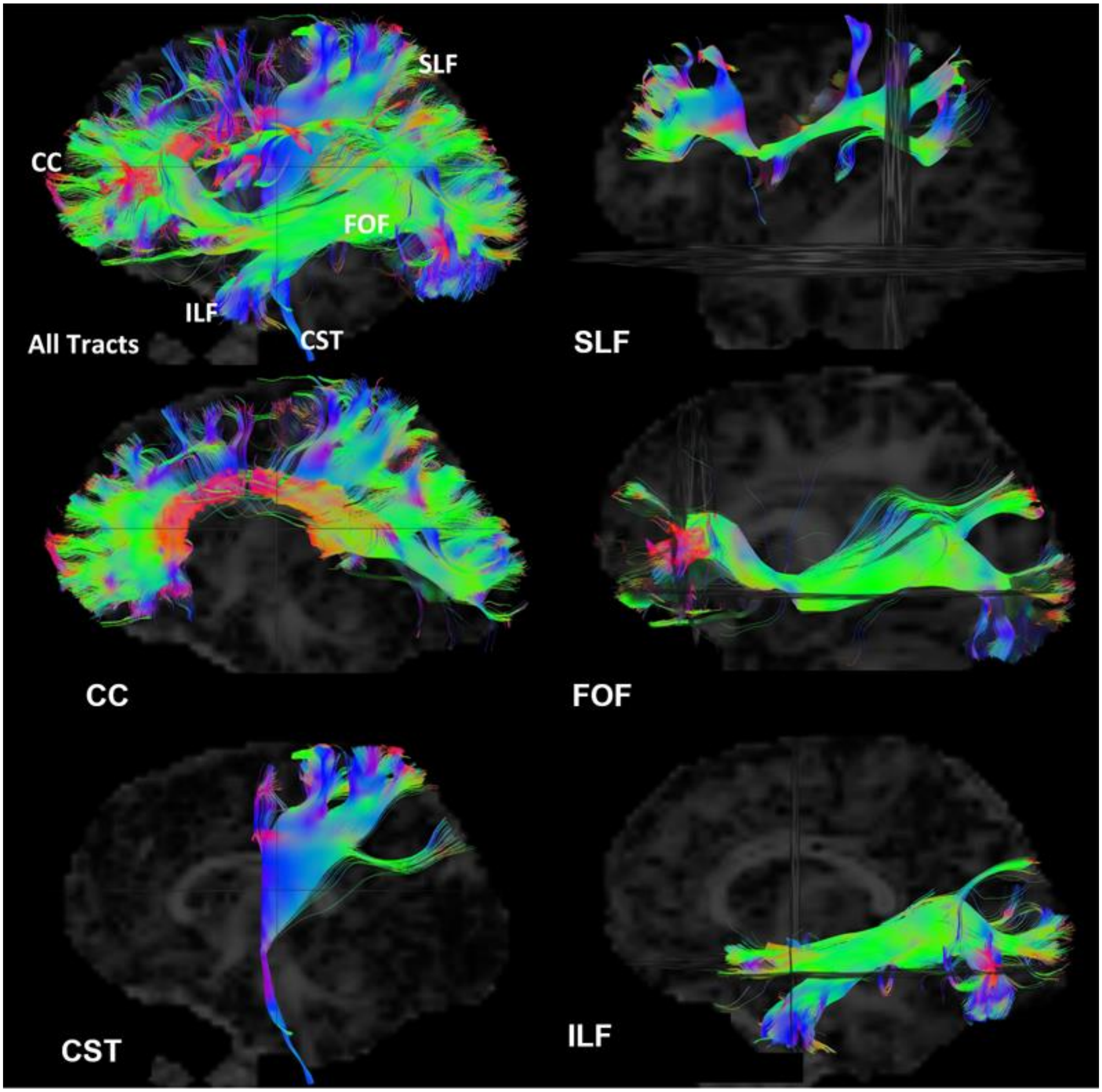

Figure S2. White Matter Tracts Generated by Tractography.
This figure illustrates the white matter tracts generated with an in-house automated tractography pipeline built upon DSI studio. The corpus callosum (CC) is divided into genu, body, and splenium. The cortico-spinal tract (CST), fronto-occipital fasciculus (FOF), inferior longitudinal fasciculus (ILF), and superior longitudinal fasciculus (SLF) were divided into left and right tracts.

Suppelemental Methods WMI segmentation

All MRI series (Scan 1, Scan 2 and Scan 3) underwent review by a pediatric neuroradiologist (J.V.H.) to assess for the presence of brain injury. An "injury" was defined as the presence of any hemorrhagic stroke, ischemic stroke, or white matter injury (lesions were defined as significant if observed injury > 1 mm). For the cases with large enough lesion volume, segmentation was carried out using methods previously described by our group.[11]

Suppelemental Methods Statistical Analysis:

Multivariable-model Covariate Selection

The selection of covariates are as follows. The thirteen non-imaging risk factors were examined and include: socio-demographic factors consisting of parental SES, maternal IQ, sex, and race & ethnicity; factors intrinsic to the participant consisting of the presence of genetic abnormalities, 22q deletion, and number of cardiac ventricles; perinatal factors consisting of gestational age at birth and birth weight; medical care and surgery related factors consisting of whether the patient had open sternum surgery, number of days with open sternum, and length of stay at the hospital; and white matter injury. An initial univariable regression analysis was conducted between each risk factor and each of the neurodevelopmental tests. From this initial regression test, the risk factors that demonstrated significant associations (alpha < 0.05) with outcomes are processed through correlation tests for collinearity and then subjected to model selection. This screening multi-variable model selection is composed of non-imaging factors only – without imaging data – and is used as a model reduction to eliminate the predictor variables that are not contributory. The factors that were found to be consistently associated with tests performance within this multi-variable regression screen were incorporated into the final model.

Thus, we investigated the relationship of each imaging trajectory to each neurodevelopmental test using the multi-variable analysis with a fixed set of non-imaging variables as covariates. The results from this model selection process are presented in eResults of this Supplement.

Supplemental Methods Factor Contribution Analysis

$$Factor\ Contribution\ of\ Variable = \frac{PRE\ of\ Variable}{PRE\ Total} \times R^2\ (Equation\ S1)$$

Equation S1. Factor Contribution Analysis calculation.

After the multi-variable regression analysis, a post-hoc factor contribution analysis was conducted for each model that demonstrated significant (variable specific $p<0.05$) association between imaging trajectory and neuropsychological test. This post-hoc analysis examined the contributions of each independent variable – imaging, as well as the non-imaging in the final multi-variable model – to the variance in test performance using the formula presented in Equation S1.

Supplemental Results

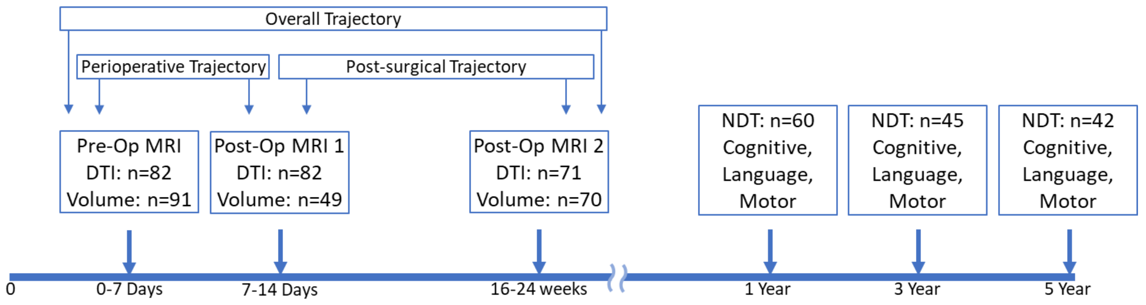

Figure S3. Study Design and Cohort of Participants Imaging and Neurodevelopmental Testing Timeline.
Overview of imaging and neuropsychological testing time points, along with imaging trajectory scheme. Perioperative Trajectory is between Pre-Op MRI and Post-Op MRI #1. Post-surgical Trajectory is from Post-Op MRI#1 to Post-Op MRI#2. Overall Trajectory encompasses the Pre-Op MRI to Post-Op MRI#2. Neuropsychological tests assessing cognitive, language, and motor were conducted with Bayley-III for one and three years. For five year assessment WPPSI-III Full Scale IQ, WPSSI-III Verbal IQ, and Beery VMI were used for cognitive, language, and motor, respectively.

Supplemental Results Summary Statistics

The summary statistics of Brain Volumes are presented in **Table S2.** The summary statistics of White Matter Tractography FA and RD measurements are presented in Table S3. The summary statistics of Neurodevelopmental Tests (NDT) Scores are presented in Table S4. The summary statistics of Brain Volumes trajectories – given as changes in cubic centimeters over weeks – are presented in Table S5. The summary statistics of DTI FA and RD tractography measurements – given as change in FA index and 10^{-3}mm2/s/week– are presented in Table S6 and S7, respectively.

Table S2. Brain Volumes Summary Statistics

	Pre-Op (N = 91)	Post-Op1 (N = 39)	Post-Op2 (N = 70)
Volumes (in cc)	Mean (SD)	Mean (SD)	Mean (SD)
Brainstem	5289 (674)	674 (5294)	5294 (5343)
Cerebellum	24920 (3100)	3100 (24146)	24146 (24721)
Cortex	187931 (30171)	30171 (197105)	197105 (192434)
CSF	76828 (26167)	26167 (77355)	77355 (90893)
DGM	20374 (4588)	4588 (20594)	20594 (20320)
WM	150766 (24147)	24147 (145428)	145428 (151057)
Whole Brain with CSF	469526 (45068)	45068 (472092)	472092 (479494)
Whole Brain without CSF	392698 (43714)	43714 (392801)	392801 (388601)

Abbreviations: DGM - deep grey matter; WM - white matter, cc – cubic centimeter

Table S3. White Matter Tract DTI FA and RD Summary Statistics

	Pre-Op (N = 82)	Post-Op1 (N = 82)	Post-Op2 (N = 71)
Fractional Anisotropy	Mean (SD)	Mean (SD)	Mean (SD)
CC Genu	0.2359 (0.0207)	0.2371 (0.0215)	0.3053 (0.0269)
CC Body	0.2117 (0.0194)	0.208 (0.0211)	0.265 (0.0242)
CC Splenium	0.257 (0.0235)	0.2569 (0.0197)	0.3255 (0.0378)
CST-L	0.2552 (0.0244)	0.2597 (0.0241)	0.3502 (0.0323)
CST-R	0.2431 (0.023)	0.2421 (0.0223)	0.3407 (0.0368)
FOF-L	0.2057 (0.0187)	0.209 (0.0185)	0.2719 (0.0248)
FOF-R	0.2064 (0.0162)	0.2097 (0.019)	0.2718 (0.0242)
ILF-L	0.1877 (0.0167)	0.1938 (0.02)	0.2662 (0.0244)
ILF-R	0.1963 (0.0172)	0.1994 (0.0191)	0.2672 (0.0232)
SLF-L	0.186 (0.0201)	0.1879 (0.0275)	0.2394 (0.0246)
SLF-R	0.1787 (0.0237)	0.1833 (0.0246)	0.238 (0.023)
Radial Diffusivity (10-3mm2/s)			
CC Genu	1.2457 (0.0846)	1.2381 (0.082)	0.9816 (0.0862)
CC bBody	1.2697 (0.1092)	1.288 (0.1188)	1.0589 (0.1106)
CC Splenium	1.2303 (0.11)	1.2298 (0.0926)	1.0286 (0.1557)
CST-L	1.0804 (0.0856)	1.0796 (0.1034)	0.8782 (0.0837)
CST-R	1.1069 (0.1326)	1.1223 (0.1154)	0.8913 (0.086)
FOF-L	1.2188 (0.073)	1.2051 (0.0805)	0.9748 (0.0665)
FOF-R	1.212 (0.069)	1.196 (0.0808)	0.9487 (0.0624)
ILF-L	1.296 (0.0832)	1.2963 (0.0978)	1.0078 (0.1172)
ILF-R	1.2736 (0.0841)	1.2597 (0.0819)	0.9679 (0.0713)
SLF-L	1.1615 (0.0911)	1.1768 (0.1044)	0.9328 (0.0724)
SLF-R	1.1941 (0.1052)	1.1823 (0.1055)	0.95 (0.0979)

Abbreviations: CC – Corpus Callosum; CST – Cortical Spinal Tract; FOF – Fronto-Occipital Fasciculus; ILF – Inferior Longitudinal Fasciculus; SLF – Superior Longitudinal Fasciculus; L – Left; R – Right

Table S4. Neurodevelopmental Tests Summary Statistics

Neurodevelopmental Tests	Mean (SD)
5-Year NDT (N=36)	
WPPSI-III Verbal IQ	94.98 (15.42)
WPPSI-III Full Scale IQ	96.1 (17.7)
Beery-VMI Motor	85.48 (14.25)
3-Year NDT (N=39)	
Bayley-III Language	95.07 (12.45)
Bayley-III Cognitive	97.07 (10.3)
Bayley-III Motor	98.89 (12.48)
1-Year NDT (N=54)	
Bayley-III Language	88 (12.56)
Bayley-III Cognitive	101.25 (13.49)
Bayley-III Motor	89.14 (14.19)

Abbreviations: 5-Year NDT – 5-Year Neurodevelopmental Testing Outcome; 3-Year NDT – 3-Year Neurodevelopmental Testing Outcome; 1-Year NDT – 1-Year Neurodevelopmental Testing Outcome.

Table S5. Brain Volume Trajectories Summary Statistics

Units: cc/week	Year 1	Year 3	Year 5
	Mean (SD)	Mean (SD)	Mean (SD)
Early Infant Trajectory	*(N=54)*	*(N=39)*	*(N=36)*
Brainstem	177 (51)	183 (52)	183 (52)
Cerebellum	1571 (482)	1608 (478)	1611 (512)
Cortex	8702 (2684)	8957 (2663)	8817 (2491)
CSF	3823 (3257)	4150 (3761)	4099 (3714)
DGM	867 (384)	957 (404)	967 (396)
WM	4761 (1892)	4810 (1935)	4842 (1718)
Whole Brain with CSF	18038 (3469)	18247 (3755)	18323 (3577)
Whole Brain without CSF	14216 (4015)	14097 (4277)	14224 (3832)
Post-surgical Trajectory	*(N=50)*	*(N=37)*	*(N=36)*
Brainstem	200 (65)	213 (58)	206 (73)
Cerebellum	1850 (515)	1922 (488)	1901 (523)
Cortex	9713 (2714)	9521 (2665)	9964 (2258)
CSF	4124 (3414)	4651 (3833)	4825 (3954)
DGM	933 (398)	1013 (395)	1017 (416)
WM	5881 (2315)	6436 (1795)	6280 (1855)
Whole Brain with CSF	20433 (3164)	21093 (2854)	21203 (3088)
Whole Brain without CSF	16308 (3901)	16442 (3934)	16377 (4147)
Perioperative Trajectory	*(N=48)*	*(N=36)*	*(N=36)*
Brainstem	93 (567)	-10 (458)	160 (578)
Cerebellum	-92 (2392)	-563 (1862)	-439 (1725)
Cortex	5447 (33022)	6001 (29748)	443 (27984)
CSF	5867 (30933)	-3443 (27405)	-1522 (29547)
DGM	608 (3671)	882 (3623)	619 (3624)
WM	-832 (21705)	-6032 (19698)	-5069 (16936)
Whole Brain with CSF	7823 (22258)	1152 (20170)	1103 (20225)
Whole Brain without CSF	1956 (35529)	4595 (32811)	2624 (28305)

Table S6. White Matter Tract DTI FA Trajectories Summary Statistics

Units: FA Index/week	Year 1	Year3	Year5
	Mean (SD)	Mean (SD)	Mean (SD)
Early Infant Trajectory	*(N=54)*	*(N=39)*	*(N=36)*
CCBody	0.0024 (0.0011)	0.0024 (0.0012)	0.0025 (0.0011)
CST-L	0.0047 (0.0015)	0.0047 (0.0015)	0.0047 (0.0016)
CST-R	0.0047 (0.0018)	0.0046 (0.0019)	0.0046 (0.0019)
FOF-L	0.003 (0.0011)	0.0029 (0.0011)	0.003 (0.0011)
FOF-R	0.0031 (0.0012)	0.0029 (0.0013)	0.0031 (0.0013)
Genu	0.0034 (0.001)	0.0034 (0.0011)	0.0033 (0.0011)
ILF-L	0.0038 (0.0013)	0.0037 (0.0013)	0.0038 (0.0014)
ILF-R	0.0036 (0.0013)	0.0035 (0.0012)	0.0036 (0.0014)
SLF-L	0.0023 (0.0012)	0.0024 (0.001)	0.0022 (0.0011)
SLF-R	0.0029 (0.0016)	0.003 (0.0019)	0.0032 (0.0018)
Splenium	0.0031 (0.0014)	0.0028 (0.0017)	0.0032 (0.0016)
Post-surgical Trajectory	*(N=50)*	*(N=37)*	*(N=36)*
CCBody	0.0028 (0.0011)	0.003 (0.0011)	0.003 (0.0011)
CST-L	0.0046 (0.0017)	0.0045 (0.0017)	0.0046 (0.0015)
CST-R	0.0051 (0.0021)	0.0053 (0.0021)	0.0051 (0.002)
FOF-L	0.0031 (0.0013)	0.003 (0.0012)	0.0031 (0.0013)
FOF-R	0.0031 (0.0011)	0.003 (0.001)	0.0032 (0.001)
Genu	0.0036 (0.0013)	0.0037 (0.0012)	0.0034 (0.0012)
ILF-L	0.0035 (0.0014)	0.0034 (0.0013)	0.0033 (0.0014)
ILF-R	0.0036 (0.0014)	0.0037 (0.0014)	0.0037 (0.0015)
SLF-L	0.0025 (0.0015)	0.0028 (0.0014)	0.0026 (0.0013)
SLF-R	0.0032 (0.0017)	0.0031 (0.0017)	0.003 (0.0017)
Splenium	0.0033 (0.0013)	0.0031 (0.0015)	0.0033 (0.0015)
Perioperative Trajectory	*(N=48)*	*(N=36)*	*(N=36)*
CCBody	-0.0033 (0.0169)	-0.003 (0.0147)	-0.0027 (0.0162)
CST-L	0.0035 (0.0231)	0.003 (0.02)	0.0027 (0.0212)
CST-R	-0.0012 (0.0246)	-0.0036 (0.0236)	-0.0066 (0.023)
FOF-L	0.0032 (0.0176)	0.0036 (0.0095)	0.0015 (0.0102)
FOF-R	0.0028 (0.0161)	0.0018 (0.0081)	0.0007 (0.008)
Genu	0.0007 (0.0175)	-0.0019 (0.0139)	0.0011 (0.0166)
ILF-L	0.0061 (0.0165)	0.0054 (0.0119)	0.006 (0.0118)
ILF-R	0.0037 (0.015)	0.0011 (0.0085)	0.0019 (0.01)
SLF-L	0.0018 (0.0287)	-0.0063 (0.0216)	-0.0039 (0.0215)
SLF-R	0.0011 (0.0233)	0.0064 (0.0188)	0.0118 (0.0197)
Splenium	0.0003 (0.0175)	0.0007 (0.0193)	0.0002 (0.0175)

Table S7. White Matter Tract DTI RD Trajectories Summary Statistics

10^{-3}mm/s/week	Year 1	Year3	Year5
	Mean (SD)	Mean (SD)	Mean (SD)
Early Infant Trajectory	*(N=54)*	*(N=39)*	*(N=36)*
CCBody	-0.0102 (0.0056)	-0.0097 (0.0046)	-0.0099 (0.0062)
CST-L	-0.0101 (0.0053)	-0.01 (0.006)	-0.0104 (0.0058)
CST-R	-0.0109 (0.0102)	-0.0111 (0.0116)	-0.0111 (0.0115)
FOF-L	-0.0119 (0.0036)	-0.0119 (0.0036)	-0.0118 (0.0037)
FOF-R	-0.0132 (0.0034)	-0.0127 (0.0032)	-0.0124 (0.0034)
Genu	-0.0133 (0.0044)	-0.0133 (0.004)	-0.0129 (0.0049)
ILF-L	-0.0147 (0.0059)	-0.0147 (0.0063)	-0.0143 (0.0065)
ILF-R	-0.0152 (0.0039)	-0.0152 (0.004)	-0.0153 (0.0043)
SLF-L	-0.0106 (0.0047)	-0.0095 (0.0047)	-0.0097 (0.0048)
SLF-R	-0.0122 (0.0053)	-0.0126 (0.0055)	-0.0133 (0.0049)
Splenium	-0.0108 (0.0053)	-0.0098 (0.0051)	-0.0106 (0.0061)
Post-surgical Trajectory	*(N=50)*	*(N=37)*	*(N=36)*
CCBody	-0.012 (0.0055)	-0.0121 (0.0054)	-0.0113 (0.0056)
CST-L	-0.01 (0.0067)	-0.01 (0.0073)	-0.0102 (0.0071)
CST-R	-0.0114 (0.0067)	-0.0118 (0.0072)	-0.0117 (0.0074)
FOF-L	-0.0117 (0.0043)	-0.0116 (0.0043)	-0.0115 (0.0044)
FOF-R	-0.013 (0.0041)	-0.013 (0.0042)	-0.0129 (0.0043)
Genu	-0.0135 (0.0048)	-0.0137 (0.005)	-0.0128 (0.0052)
ILF-L	-0.0146 (0.0064)	-0.0144 (0.0064)	-0.0136 (0.0062)
ILF-R	-0.0152 (0.0045)	-0.016 (0.0044)	-0.0154 (0.0046)
SLF-L	-0.0122 (0.0056)	-0.0123 (0.0052)	-0.0106 (0.0042)
SLF-R	-0.0121 (0.0073)	-0.0126 (0.0049)	-0.0107 (0.0087)
Splenium	-0.0109 (0.0046)	-0.0101 (0.0052)	-0.0097 (0.0053)
Perioperative Trajectory	*(N=48)*	*(N=36)*	*(N=36)*
CCBody	0.0183 (0.086)	0.0209 (0.0386)	0.0097 (0.0936)
CST-L	0.0021 (0.1104)	0.01 (0.1299)	-0.0034 (0.1296)
CST-R	0.0096 (0.1345)	0.0289 (0.1525)	0.026 (0.1585)
FOF-L	-0.0159 (0.0494)	-0.0168 (0.0453)	-0.0145 (0.046)
FOF-R	-0.0139 (0.0496)	-0.009 (0.041)	-0.0086 (0.0422)
Genu	-0.0025 (0.0402)	0.0001 (0.0327)	-0.0043 (0.034)
ILF-L	0.0025 (0.0721)	-0.0015 (0.0562)	0.0102 (0.0695)
ILF-R	-0.0133 (0.0595)	-0.0021 (0.0361)	-0.0064 (0.0536)
SLF-L	0.0168 (0.0825)	0.0473 (0.0783)	0.0399 (0.0832)
SLF-R	0.0132 (0.095)	-0.0009 (0.0693)	-0.0279 (0.0654)
Splenium	0.002 (0.0944)	0.0069 (0.0478)	-0.0102 (0.0995)

Supplemental Results Non-imaging Risk Factors Covariate Selection for Multi-variable Model

Initial regression analysis was conducted between 13 non-imaging risk factors and NDT outcomes, and the results are presented in separate tables as patient soci-demographics (**Table S8**), intrinsic factors (**Table S9**), perinatal factors (**Table S10**), medical care and surgery related factors (**Table S11**), and white matter injury (**Table S12**).Maternal IQ demonstrated the most significant of all the risk factors, with all 9 findings were significant ($p < 0.05$). Presence of genetic abnormality has the second most significant associations with 8 significant findings. These two factors are associated with poor performance on cognitive, language, and motor tests, and consistent throughout all three NDT epochs. Parental SES showed significant correlation with cognitive, language, and motor in infancy, and cognitive and language in 3-year early childhood NDT. SES seems to be less of a factor in a child's performance as they age, as there were no correlations to 5-Year early childhood NDT. Length of hospital stay and ethnicity were found to correlate with infant NDT outcomes but were largely not correlated with early childhood assessment. Number of cardiac ventricles and sex had one significant correlation. Birthweight, along with whether the participant had open sternum and the duration of the open sternum had no correlations.

To test whether these environmental and clinical risk factors were independent or whether there were overlaps between them, a correlation analysis among these non-imaging variables was conducted (**Table S13**) to test for collinearity. Maternal IQ and Parental SES had a significantly high correlation (Pearson's $R = 0.6798$) indicating that these two factors were colinear. Parental SES and Maternal IQ were also correlated with ethnicity and race, indicating multicollinearities between these socio-demographic factors.

After further examination of the ethnicity and race data revealed that this variable was not clearly separable since the attrition of Black and Asian participants in the 3- and 5-year NDT leading to two and zero participants, respectively. This observation and the collinearity of ethnicity with SES, as well as the fact that Ethnicity and race had the least significant findings in the univariable regression with NDT (**Table S8**) lead to its removal from consideration from the multi-variable model.

Table S8. Comparison of Neurodevelopmental Outcomes to Non-imaging Factors – Socio-Demographic

Neurodevelopmental Tests	Parental SES		Maternal IQ	
	β Coefficient (95% CI)	p-value	β Coefficient (95% CI)	p-value
5-Year NDT				
WPPSI-III Verbal IQ	0.352 (-0.015 - 0.719)	0.0595	0.609 (0.32 - 0.897)	**0.0001**
WPPSI-III Full Scale IQ	0.245 (-0.19 - 0.68)	0.2606	0.679 (0.342 - 1.016)	**0.0002**
Beery-VMI Motor	0.094 (-0.218 - 0.406)	0.5466	0.35 (0.105 - 0.596)	**0.0064**
3-Year NDT				
Bayley-III Language	0.492 (0.265 - 0.719)	**<.0001**	0.414 (0.226 - 0.601)	**<.0001**
Bayley-III Cognitive	0.271 (0.059 - 0.482)	**0.0134**	0.332 (0.176 - 0.489)	**0.0001**
Bayley-III Motor	0.158 (-0.11 - 0.426)	0.2407	0.242 (0.028 - 0.457)	**0.0277**
1-Year NDT				
Bayley-III Language	0.339 (0.109 - 0.569)	**0.0047**	0.276 (0.091 - 0.461)	**0.0042**
Bayley-III Cognitive	0.324 (0.086 - 0.561)	**0.0086**	0.302 (0.102 - 0.503)	**0.0039**
Bayley-III Motor	0.261 (0.009 - 0.514)	**0.0427**	0.275 (0.063 - 0.486)	**0.0118**
	Sex		Ethnicity	
	β Coefficient (95% CI)	p-value	β Coefficient (95% CI)	p-value
5-Year NDT				
WPPSI-III Verbal IQ	-8.071 (-17.824 - 1.681)	0.1021	7.226 (-4.562 - 19.013)	0.2224
WPPSI-III Full Scale IQ	-8.064 (-19.505 - 3.378)	0.1618	5.738 (-8.036 - 19.513)	0.4043
Beery-VMI Motor	-1.514 (-10.592 - 7.564)	0.7379	2.65 (-7.273 - 12.573)	0.5924
3-Year NDT				
Bayley-III Language	-5.728 (-13.442 - 1.985)	0.1415	5.503 (-3.146 - 14.152)	0.2063
Bayley-III Cognitive	-4.662 (-11.045 - 1.722)	0.1481	6.345 (-0.677 - 13.366)	0.0754
Bayley-III Motor	-7.586 (-15.161 - -0.011)	**0.0497**	5.027 (-3.665 - 13.719)	0.2499
1-Year NDT				
Bayley-III Language	1.319 (-5.357 - 7.995)	0.6939	9.673 (2.974 - 16.373)	**0.0054**
Bayley-III Cognitive	1.286 (-5.836 - 8.408)	0.7191	9.722 (2.49 - 16.955)	**0.0093**
Bayley-III Motor	1.5 (-6.039 - 9.039)	0.6918	13.786 (6.555 - 21.016)	**0.0003**

Table S9. Comparison of Neurodevelopmental Outcomes to Non-imaging Intrinsic Factors

Neurodevelopmental Tests	Presence of Genetic Abnormalities		Genetics: 22q deletion		Number of Cardiac Ventricles	
	β Coefficient (95% CI)	p-value	β Coefficient (95% CI)	p-value	β Coefficient (95% CI)	p-value
5-Year NDT						
WPPSI-III Verbal IQ	12.823 (2.009 - 23.636)	**0.0213**	-4.583 (-21.226 - 12.06)	0.5431	2.48 (-7.584 - 12.545)	0.6209
WPPSI-III Full Scale IQ	15.344 (2.387 - 28.302)	**0.0215**	-5.1 (-28.198 - 17.998)	0.6177	1.292 (-10.551 - 13.135)	0.8264
Beery-VMI Motor	13.051 (2.91 - 23.191)	**0.013**	6.167 (-24.165 - 36.498)	0.6454	-1.028 (-10.113 - 8.057)	0.8203
3-Year NDT						
Bayley-III Language	9.636 (1.601 - 17.672)	**0.0199**	3.333 (-13.881 - 20.548)	0.6753	0.679 (-6.91 - 8.267)	0.8577
Bayley-III Cognitive	8.159 (1.536 - 14.782)	**0.0169**	-2.833 (-11.937 - 6.271)	0.5038	-3.429 (-9.617 - 2.76)	0.2701
Bayley-III Motor	10.985 (3.099 - 18.871)	**0.0074**	-0.667 (-13.938 - 12.605)	0.9131	-7.917 (-15.121 - -0.712)	**0.032**
1-Year NDT						
Bayley-III Language	8.892 (1.031 - 16.752)	**0.0273**	5.167 (-11.452 - 21.785)	0.5042	-0.751 (-7.379 - 5.876)	0.8213
Bayley-III Cognitive	7.813 (-0.732 - 16.357)	0.0724	0 (-23.012 - 23.012)	1	-4.688 (-11.625 - 2.25)	0.1815
Bayley-III Motor	10.213 (1.349 - 19.076)	**0.0247**	-1 (-20.154 - 18.154)	0.9097	-2.299 (-9.762 - 5.165)	0.5399

Table S10. Comparison of Neurodevelopmental Outcomes to Non-imaging Perinatal Factors

Neurodevelopmental Tests	Gestational Age at Birth		Birth Weight	
	β Coefficient (95% CI)	p-value	β Coefficient (95% CI)	p-value
5-Year NDT				
WPPSI-III Verbal IQ	-1.158 (-4.724 - 2.409)	0.5154	0.004 (-0.007 - 0.014)	0.4641
WPPSI-III Full Scale IQ	-1.081 (-5.208 - 3.046)	0.5991	0.005 (-0.007 - 0.017)	0.3936
Beery-VMI Motor	-1.284 (-4.615 - 2.046)	0.4404	0.001 (-0.008 - 0.011)	0.7664
3-Year NDT				
Bayley-III Language	-0.111 (-3.325 - 3.104)	0.9448	0 (-0.009 - 0.008)	0.9562
Bayley-III Cognitive	0.75 (-1.907 - 3.408)	0.5719	0.004 (-0.003 - 0.011)	0.215
Bayley-III Motor	0.355 (-2.892 - 3.602)	0.8264	0.005 (-0.003 - 0.014)	0.2061
1-Year NDT				
Bayley-III Language	0.779 (-1.946 - 3.504)	0.5693	0.001 (-0.005 - 0.008)	0.6608
Bayley-III Cognitive	0.206 (-2.72 - 3.132)	0.8883	-0.001 (-0.008 - 0.006)	0.767
Bayley-III Motor	0.564 (-2.519 - 3.647)	0.7156	-0.001 (-0.008 - 0.007)	0.8312

Table S11. Comparison of Neurodevelopmental Outcomes to Medical Care & Surgery Factors

Neurodevelopmental Tests	Hospital Length of Stay (days)		Had Open Sternum		Number of Days with Open Sternum	
	β Coefficient (95% CI)	p-value	β Coefficient (95% CI)	p-value	β Coefficient (95% CI)	p-value
5-Year NDT						
WPPSI-III Verbal IQ	-0.096 (-0.365 - 0.173)	0.4756	-3.038 (-16.221 - 10.145)	0.6437	5.962 (-8.836 - 20.759)	0.3479
WPPSI-III Full Scale IQ	-0.08 (-0.446 - 0.285)	0.6593	0.069 (-16.19 - 16.327)	0.9932	6.7 (-13.145 - 26.545)	0.4016
Beery-VMI Motor	-0.153 (-0.394 - 0.088)	0.2068	7.691 (-3.499 - 18.881)	0.1725	1.484 (-25.074 - 28.042)	0.8957
3-Year NDT						
Bayley-III Language	-0.106 (-0.222 - 0.01)	0.0725	2.529 (-6.548 - 11.605)	0.5772	8.25 (-2.611 - 19.111)	0.1179
Bayley-III Cognitive	-0.094 (-0.189 - 0.001)	0.0524	3.3 (-4.165 - 10.765)	0.3776	4.286 (-6.716 - 15.287)	0.3952
Bayley-III Motor	-0.131 (-0.245 - -0.017)	**0.0248**	4.871 (-4.131 - 13.873)	0.2812	5.036 (-8.285 - 18.356)	0.4087
1-Year NDT						
Bayley-III Language	-0.116 (-0.224 - -0.008)	**0.0354**	-0.602 (-9.405 - 8.201)	0.8916	4.821 (-5.811 - 15.453)	0.3262
Bayley-III Cognitive	-0.164 (-0.276 - -0.052)	**0.0049**	-2.1 (-11.516 - 7.316)	0.6569	4.643 (-12.198 - 21.483)	0.5427
Bayley-III Motor	-0.158 (-0.278 - -0.039)	**0.0104**	5.702 (-4.126 - 15.53)	0.2502	-1.286 (-20.304 - 17.732)	0.88

Table S12. Comparison of Neurodevelopmental Outcomes to Non-imaging Factors – White Matter Injury

Neurodevelopmental Tests	WMI	
	β Coefficient (95% CI)	p-value
5-Year NDT		
WPPSI-III Verbal IQ	7.033 (-2.081 - 16.146)	0.1265
WPPSI-III Full Scale IQ	6.839 (-3.992 - 17.671)	0.2087
Beery-VMI Motor	4.095 (-4.661 - 12.852)	0.35
3-Year NDT		
Bayley-III Language	3.323 (-3.252 - 9.898)	0.3136
Bayley-III Cognitive	1.963 (-4.219 - 8.145)	0.5252
Bayley-III Motor	2.161 (-5.419 - 9.742)	0.5681
1-Year NDT		
Bayley-III Language	2.004 (-4.607 - 8.614)	0.5462
Bayley-III Cognitive	1.561 (-5.577 - 8.699)	0.6631
Bayley-III Motor	2.951 (-4.616 - 10.518)	0.4379

Table S13. Correlation Among Non-Imaging Factors

		Maternal IQ	Cardiac Ventricles	Sex	Birth weight	Hospital LoS	ethnicity	Race	Genetic Abnormality
**Parental SES**	Pearson R	0.6798	0.0287	0.1793	-0.0522	-0.2205	-0.3057	0.1957	0.1477
	p-value	**<.0001**	0.8291	0.1743	0.6945	0.0934	**0.0185**	0.1375	0.2643
**Maternal IQ**	Pearson R		-0.0426	0.0871	0.0561	-0.1294	-0.4928	0.29	0.109
	p-value		0.7339	0.4866	0.6545	0.3005	**<.0001**	**0.0182**	0.3835
**Cardiac Ventricles**	Pearson R			-0.0705	0.1912	-0.3768	0.0009	-0.0114	0.1474
	p-value			0.497	0.0649	**0.0002**	0.9929	0.9129	0.1539
**Sex**	Pearson R				0.1514	-0.0034	-0.0994	-0.0544	-0.0848
	p-value				0.1408	0.9738	0.3329	0.5964	0.414
**Birth weight**	Pearson R					-0.1408	-0.0639	0.0784	-0.0451
	p-value					0.1713	0.5365	0.4476	0.6661
**Hospital LoS**	Pearson R						0.2459	-0.0005	0.2052
	p-value						**0.0152**	0.9961	**0.046**
**ethnicity**	Pearson R							0.2612	0.0221
	p-value							**0.0098**	0.8316
**race**	Pearson R								0.1799
	p-value								0.0811

Abbreviations: Parental SES – Parental Socioeconomic Statusl Hospital LoS – Hospital Length of Stay

Supplemental Results Non-imaging Risk Factors Covariate Selection for Multi-variable Model Adjusted Regression

Based on the findings thus far (**Tables S8-S13**), it was determined that Maternal IQ, Parental SES, presence of genetic abnormality, and length of hospital stay maybe significant contributors to NDT variance, and they were considered as possible covariates for inclusion in the multi-variable model comparing imaging trajectories to NDT outcomes. While number of cardiac ventricles did not show high level of correlation to NDT on its own, this factor modeled for the nature of CHD lesion and was also considered for inclusion. Consequently, two competing multi-variable regression analyses were run to determine whether to include Maternal IQ or Parental SES. Due to the high collinearity between Maternal IQ and Parental SES, it was deemed the two factors are not independent and including both in the same model would be redundant. Each multivariable model had Maternal IQ or Parental SES and included the other factors of interest as covariates – number of cardiac ventricles, length of hospital stay, and presence of genetic abnormality.

The results from these two competing analyses (**Tables S14 and S15**) clearly demonstrate that in the multi-variable model, in the presence of all the other non-imaging factors, Maternal IQ retained significant associations with most of the neurocognitive tests, while Parental SES was only associated with two of the neurocognitive tests. Length of Hospital Stay was also found to not be associated in the presence of other factors. Based on this examination, Maternal IQ was chosen for the final multivariate model, and length of hospital stay and Parental SES were not included.

Table S14. Maternal IQ Multi-variable Regression

Neurodevelopmental Tests	Maternal IQ		Number of Cardiac Ventricles		Presence of Genetic Abnormalities		Hospital Length of Stay (days)	
	β Coefficient (95% CI)	p-value	β Coefficient (95% CI)	p-value	β Coefficient (95% CI)	p-value	β Coefficient (95% CI)	p-value
5-Year NDT								
WPPSI-III Verbal IQ	0.571 (0.281 - 0.861)	**0.0003**	-1.166 (-10.708 - 8.376)	0.8054	11.044 (0.803 - 21.285)	**0.0353**	0.024 (-0.222 - 0.27)	0.8425
WPPSI-III Full Scale IQ	0.65 (0.31 - 0.99)	**0.0005**	-4.483 (-15.609 - 6.642)	0.4182	14.005 (1.705 - 26.305)	**0.0269**	0.082 (-0.24 - 0.404)	0.6087
Beery-VMI Motor	0.331 (0.096 - 0.566)	**0.0071**	-3.053 (-12.231 - 6.126)	0.504	12.49 (2.056 - 22.924)	**0.0204**	-0.044 (-0.285 - 0.197)	0.7115
3-Year NDT								
Bayley-III Language	0.413 (0.239 - 0.587)	**<.0001**	-0.826 (-7.309 - 5.656)	0.798	8.958 (2.061 - 15.855)	**0.0122**	-0.064 (-0.167 - 0.04)	0.2204
Bayley-III Cognitive	0.352 (0.216 - 0.489)	**<.0001**	-5.526 (-10.614 - -0.438)	**0.034**	8.882 (3.469 - 14.295)	**0.0019**	-0.027 (-0.107 - 0.054)	0.5117
Bayley-III Motor	0.28 (0.104 - 0.456)	**0.0026**	-10.231 (-16.8 - -3.663)	**0.0031**	12.532 (5.543 - 19.52)	**0.0008**	-0.027 (-0.131 - 0.078)	0.6056
1-Year NDT								
Bayley-III Language	0.284 (0.105 - 0.463)	**0.0025**	-0.476 (-7.332 - 6.379)	0.8896	9.594 (1.702 - 17.486)	**0.0182**	-0.065 (-0.181 - 0.05)	0.263
Bayley-III Cognitive	0.309 (0.117 - 0.501)	**0.0022**	-3.803 (-11.159 - 3.552)	0.3042	9.31 (0.842 - 17.778)	**0.0318**	-0.084 (-0.208 - 0.04)	0.1797
Bayley-III Motor	0.276 (0.075 - 0.477)	**0.008**	-1.031 (-8.732 - 6.669)	0.7892	10.49 (1.624 - 19.355)	**0.0213**	-0.101 (-0.231 - 0.028)	0.1224

Table S15. Parental SES Multivariable Regression

Neurodevelopmental Tests	Parental SES		Number of Cardiac Ventricles		Presence of Genetic Abnormalities		Hospital Length of Stay (days)	
	β Coefficient (95% CI)	p-value	β Coefficient (95% CI)	p-value	β Coefficient (95% CI)	p-value	β Coefficient (95% CI)	p-value
5-Year NDT								
WPPSI-III Verbal IQ	0.326 (-0.031 - 0.683)	0.0722	-0.065 (-10.752 - 10.622)	0.9903	13.341 (1.842 - 24.839)	**0.0243**	-0.018 (-0.294 - 0.257)	0.8928
WPPSI-III Full Scale IQ	0.231 (-0.19 - 0.652)	0.2729	-2.193 (-14.889 - 10.502)	0.7274	17.136 (3.128 - 31.143)	**0.018**	-0.028 (-0.39 - 0.335)	0.8778
Beery-VMI Motor	0.053 (-0.236 - 0.342)	0.7134	1.596 (-7.591 - 10.784)	0.7262	13.173 (3.009 - 23.338)	**0.0126**	-0.116 (-0.353 - 0.121)	0.3257
3-Year NDT								
Bayley-III Language	0.463 (0.243 - 0.683)	**0.0001**	-1.85 (-8.986 - 5.286)	0.6025	9.241 (1.369 - 17.113)	**0.0226**	-0.03 (-0.143 - 0.084)	0.6004
Bayley-III Cognitive	0.249 (0.045 - 0.454)	**0.0184**	-4.053 (-10.687 - 2.581)	0.2235	7.212 (-0.106 - 14.53)	0.0532	-0.035 (-0.14 - 0.071)	0.5099
Bayley-III Motor	0.142 (-0.091 - 0.375)	0.2246	-10.389 (-17.943 - -2.835)	**0.0084**	11.534 (3.201 - 19.867)	**0.008**	-0.027 (-0.147 - 0.093)	0.6491
1-Year NDT								
Bayley-III Language	0.302 (0.064 - 0.539)	**0.014**	-1.17 (-8.514 - 6.174)	0.7499	5.632 (-2.934 - 14.198)	0.1923	-0.074 (-0.195 - 0.048)	0.2311
Bayley-III Cognitive	0.275 (0.036 - 0.514)	**0.0248**	-2.647 (-10.025 - 4.731)	0.4738	4.852 (-3.754 - 13.458)	0.2623	-0.102 (-0.224 - 0.021)	0.1012
Bayley-III Motor	0.192 (-0.056 - 0.441)	0.1265	-0.34 (-8.027 - 7.348)	0.9295	6.907 (-2.059 - 15.874)	0.1279	-0.127 (-0.255 - 0.001)	0.0511

Supplemental Results White Matter Injury Results

There were 46 cases identified with white matter injury. There were 35 cases with WMI in the Pre-Op imaging, 7 of which had large enough lesions for segmentation and lesion volume measurement (of these only 4 had neurodevelopmental testing). In the Post-Op1 scans 11 new cases of WMI developed while 1 of the prior cases resolved, for a total of 45 cases with post-op WMI. Of these, 17 of the cases had large enough measurable lesion volume (of these 15 had neurodevelopmental testing).

Comparison analysis between participants with and without WMI for differences in volume trajectories, DTI FA and RD trajectories, and neurodevelopmental testing are presented in **Table S16, S17, S18, and S19,** respectively. There were no significant findings of consequence. Despite the low sample size, a regression analysis between white matter lesion volumes (both pre-op and post-op) and neurodevelopmental testing outcomes (against each of the three testing time points) were conducted, but there were no significant findings.

Table S16. Brain Volume Trajectory differences between the groups with and without WMI.

	Without WMI (N=16)	With WMI (N=26)		
Volumes (cc/week)	Mean (SD)	Mean (SD)	t-value	p-value
Early Infant Trajectory				
Brainstem	171 (51)	182 (58)	-0.79	0.4327
Cerebellum	1430 (498)	1627 (475)	-1.61	0.1112
Cortex	7707 (3049)	8711 (3000)	-1.31	0.1941
CSF	3600 (3899)	4252 (4704)	-0.58	0.5645
DGM	817 (467)	892 (411)	-0.69	0.4919
WM	4343 (1856)	4978 (2382)	-1.13	0.2624
Whole Brain with CSF	17275 (3496)	18259 (3496)	-1.11	0.2716
Whole Brain without CSF	13676 (4538)	14007 (5015)	-0.27	0.7889
Post-Surgical Trajectory				
Brainstem	199 (49)	202 (71)	-0.13	0.901
Cerebellum	1659 (639)	1819 (537)	-0.74	0.4642
Cortex	7874 (3912)	9464 (2867)	-1.31	0.2
CSF	3697 (3658)	4088 (4343)	-0.25	0.8055
DGM	790 (391)	1023 (404)	-1.54	0.1345
WM	5187 (2687)	5590 (2509)	-0.42	0.6806
Whole Brain with CSF	18807 (4348)	20549 (3195)	-1.29	0.207
Whole Brain without CSF	15110 (5422)	16461 (4379)	-0.76	0.4542
Perioperative Trajectory				
Brainstem	-50 (680)	170 (480)	-1.15	0.2586
Cerebellum	510 (1804)	25 (2626)	0.58	0.5675
Cortex	10441 (22473)	5883 (34312)	0.42	0.6778
CSF	-9228 (34261)	8392 (28359)	-1.67	0.1042
DGM	1610 (3983)	-322 (4148)	1.35	0.1852
WM	-3263 (21229)	78 (23967)	-0.41	0.6819
Whole Brain with CSF	10844 (19890)	6654 (23103)	0.54	0.5915
Whole Brain without CSF	20072 (38090)	-1738 (27808)	2	0.0535

Table S17. DTI FA Trajectory differences between the groups with and without WMI.

	Without WMI (N=16)	With WMI (N=42)		
Fractional Anisotropy Trajectories (FA Index / week)	Mean (SD)	Mean (SD)	t-value	p-value
Early Infant Trajectory				
CC Genu	0.0031 (0.0014)	0.0036 (0.0012)	-1.53	0.1306
CC Body	0.002 (0.0009)	0.0027 (0.0011)	-2.72	**0.0086**
CC Splenium	0.0031 (0.0018)	0.0029 (0.0016)	0.36	0.7233
CST-L	0.0047 (0.0016)	0.0046 (0.0015)	0.21	0.8317
CST-R	0.0049 (0.002)	0.0047 (0.0019)	0.45	0.6563
FOF-L	0.0033 (0.0012)	0.0031 (0.0012)	0.52	0.6077
FOF-R	0.0031 (0.0011)	0.0032 (0.0015)	-0.24	0.8126
ILF-L	0.0038 (0.001)	0.004 (0.0014)	-0.5	0.6187
ILF-R	0.0033 (0.0012)	0.0037 (0.0016)	-0.94	0.3525
SLF-L	0.0021 (0.0014)	0.0025 (0.0013)	-1.01	0.3178
SLF-R	0.0027 (0.0011)	0.0031 (0.0021)	-0.8	0.4308
Post-Surgical Trajectory				
CC Genu	0.0032 (0.0013)	0.0037 (0.0014)	-1.53	0.131
CC Body	0.0025 (0.0009)	0.0033 (0.0013)	-2.93	**0.0049**
CC Splenium	0.0031 (0.0016)	0.0034 (0.0016)	-0.79	0.4349
CST-L	0.0045 (0.0019)	0.0047 (0.0014)	-0.42	0.6744
CST-R	0.005 (0.0023)	0.0057 (0.0022)	-1.03	0.3104
FOF-L	0.0031 (0.0013)	0.0033 (0.0015)	-0.55	0.5852
FOF-R	0.0033 (0.0012)	0.0033 (0.0012)	-0.15	0.8841
ILF-L	0.0036 (0.0014)	0.0039 (0.0015)	-0.64	0.5256
ILF-R	0.0034 (0.0012)	0.0039 (0.0016)	-1.36	0.179
SLF-L	0.0028 (0.0015)	0.0025 (0.0018)	0.48	0.6314
SLF-R	0.0029 (0.0016)	0.0028 (0.0019)	0.24	0.809
Perioperative Trajectory				
CC Genu	0.0021 (0.0156)	0.0011 (0.018)	0.26	0.7961
CC Body	-0.0037 (0.0132)	-0.0008 (0.0203)	-0.71	0.4799
CC Splenium	-0.0006 (0.0149)	0.0001 (0.018)	-0.17	0.8672
CST-L	0.0047 (0.0227)	0.0031 (0.0188)	0.31	0.7568
CST-R	0.0038 (0.0233)	-0.0052 (0.0223)	1.52	0.1332
FOF-L	0.0035 (0.0096)	0.0042 (0.0197)	-0.19	0.8465
FOF-R	0.0015 (0.0082)	0.0035 (0.0185)	-0.55	0.5846
ILF-L	0.0051 (0.0106)	0.0054 (0.018)	-0.1	0.9175
ILF-R	0.0016 (0.0085)	0.0027 (0.0179)	-0.3	0.7638
SLF-L	-0.0017 (0.0179)	0.0021 (0.0315)	-0.46	0.6511
SLF-R	-0.0003 (0.0222)	-0.0004 (0.0239)	0.01	0.9931

Table S18. DTI RD Trajectory differences between the groups with and without WMI.

	Without WMI (N=16)	With WMI (N=26)		
Radial Diffusivity Trajectories ((10-3mm2/s) /week)	Mean (SD)	Mean (SD)	t-value	p-value
Early Infant Trajectory				
CC Genu	-0.0126 (0.0041)	-0.0138 (0.0043)	1.12	0.2677
CC Body	-0.0083 (0.0057)	-0.0111 (0.0064)	1.82	0.0739
CC Splenium	-0.0086 (0.0074)	-0.0109 (0.0062)	1.3	0.1971
CST-L	-0.0095 (0.0054)	-0.011 (0.0058)	1.01	0.315
CST-R	-0.0111 (0.0104)	-0.0118 (0.0101)	0.25	0.8057
FOF-L	-0.0105 (0.0035)	-0.0129 (0.0038)	2.44	**0.0181**
FOF-R	-0.0128 (0.005)	-0.0137 (0.0033)	0.78	0.4416
ILF-L	-0.0147 (0.0054)	-0.0152 (0.0069)	0.29	0.7762
ILF-R	-0.0148 (0.0049)	-0.0158 (0.0039)	0.84	0.4039
SLF-L	-0.0114 (0.0063)	-0.0098 (0.0053)	-0.9	0.3712
SLF-R	-0.0121 (0.0074)	-0.0127 (0.0058)	0.25	0.807
Post-Surgical Trajectory				
CC Genu	-0.0128 (0.0048)	-0.0148 (0.0049)	1.61	0.1118
CC Body	-0.0111 (0.0058)	-0.0135 (0.0066)	1.52	0.1349
CC Splenium	-0.0095 (0.0059)	-0.0111 (0.0058)	1.08	0.2828
CST-L	-0.0084 (0.0068)	-0.0123 (0.0064)	2.16	**0.0357**
CST-R	-0.0098 (0.0063)	-0.0145 (0.0072)	2.42	**0.0192**
FOF-L	-0.0114 (0.0054)	-0.0129 (0.005)	1.1	0.2745
FOF-R	-0.0126 (0.0052)	-0.0138 (0.0044)	0.92	0.363
ILF-L	-0.0151 (0.0066)	-0.0157 (0.0067)	0.35	0.7293
ILF-R	-0.0146 (0.0052)	-0.0168 (0.0047)	1.61	0.1136
SLF-L	-0.0156 (0.0064)	-0.0097 (0.0042)	-3.24	**0.0027**
SLF-R	-0.0125 (0.0074)	-0.0113 (0.0088)	-0.45	0.6568
Perioperative Trajectory				
CC Genu	-0.0058 (0.0342)	-0.0035 (0.0462)	-0.24	0.8073
CC Body	0.0301 (0.0642)	0.006 (0.1001)	1.24	0.22
CC Splenium	0.012 (0.0577)	-0.0103 (0.103)	1.16	0.2515
CST-L	-0.0109 (0.1241)	0.0083 (0.0646)	-0.78	0.4365
CST-R	-0.0104 (0.1658)	0.0342 (0.1404)	-1.12	0.2671
FOF-L	-0.0016 (0.0617)	-0.0154 (0.0557)	0.95	0.344
FOF-R	-0.0115 (0.0421)	-0.0156 (0.0478)	0.36	0.7203
ILF-L	0.0001 (0.0769)	0.0131 (0.0748)	-0.71	0.4829
ILF-R	-0.0122 (0.0506)	-0.0097 (0.0654)	-0.17	0.8621
SLF-L	0.0394 (0.0829)	-0.0085 (0.0956)	1.65	0.1071
SLF-R	0.0046 (0.0871)	-0.0067 (0.116)	0.28	0.7842

Table S19. Neurodevelopmental Testing difference between those with WMI and those without WMI.

Neurodevelopmental Test	Without WMI	With WMI		
	Mean (SD)	Mean (SD)	t-value	p-value
5-Year NDT	(N=16)	(N=26)		
WPPSI-III Verbal IQ	96.438 (19.404)	94.4 (12.881)	0.41	0.6877
WPPSI-III Full Scale IQ	98.5 (21.068)	95.25 (15.369)	0.56	0.5757
Beery-VMI Motor	86.563 (16.309)	85.12 (13.293)	0.31	0.7581
3-Year NDT	(N=13)	(N=32)		
Bayley-III Language	98.615 (10.508)	93.516 (13.239)	1.23	0.2245
Bayley-III Cognitive	101 (11.958)	95.645 (9.376)	1.59	0.1189
Bayley-III Motor	103.1 (12.692)	97.484 (12.258)	1.37	0.1789
1-Year NDT	(N=23)	(N=37)		
Bayley-III Language	90.783 (13.879)	86.222 (11.492)	1.37	0.176
Bayley-III Cognitive	103 (16.702)	100.1 (11.148)	0.81	0.4214
Bayley-III Motor	91.826 (16.822)	87.417 (12.159)	1.17	0.2477

Supplemental Results Attrition in Neurodevelopmental Testing and Differences in Imaging Trajectory and Sociodemographics

There were attritions in the study cohort for neurodevelopmental testing at 1-year, with fewer in the 3-year and 5-year follow up. In order to assess if there were any significant differences in imaging trajectories between those who had neurodevelopmental testing and those without neurodevelopmental testing (and thus excluded from the main analysis), a comparison analysis using T-test was conducted. The results of the comparison analysis for imaging trajectory differences between participants with and without neurodevelopmental testing are presented in **Tables S20, S21, and S22** for brain volume trajectories, and **Tables S23, S24, and S25** for white matter tractography metrics. Cortex, cerebellum, white matter, right CST FA, left ILF FA and RD, left FOF FA, left SLF RD, and Splenium RD were found to be significantly different between the two groups. However, none of these in their respective imaging epochs were predictive of NDT outcomes at the respective neuropsychological testing time points. The only significant finding of consequence to the main analysis of this study was that deep GM volume trajectory of participants with early childhood NDT testing (both 3-year and 5-year) were higher than participants without NDT testing (**Table S20**). This finding was of consequence because the deep GM trajectory was predictive of Language performance at 3-year in the main multi-variable regression analysis.

The results of the comparison analysis for sociodemographic differences between participants with and without neurodevelopmental testing are presented in **Table S26**. No differences in SES, Maternal IQ, or race/ethnicity in people who returned for testing and who did not return. Lastly, the 1-year and 3-year Neurodevelopmental differences between Participants that Attended Five-year Assessment and Those Who Did Not are presented in **Table S27**. No differences in NDT at 1-year and 3-year between participants who completed 5-year assessment and participants who did not attend 5-year assessment.

Table S20. Early Infant Volume Trajectory Differences between Groups with and without Neurodevelopmental Testing.

	Year 1 Attrition				Year 3 Attrition				Year 5 Attrition			
	Without NDT (N=25)	With NDT (N=54)			Without NDT (N=38)	With NDT (N=39)			Without NDT (N=37)	With NDT (N=36)		
	Mean (SD)	Mean (SD)	t-value	p-value	Mean (SD)	Mean (SD)	t-value	p-value	Mean (SD)	Mean (SD)	t-value	p-value
Brainstem	184 (76)	177 (51)	0.3745	0.7093	171 (61)	183 (52)	0.8768	0.3838	172 (61)	183 (52)	0.7426	0.4604
Cerebellum	1487 (545)	1571 (482)	0.5761	0.5665	1485 (504)	1608 (478)	1.0239	0.3096	1481 (452)	1611 (512)	1.0867	0.2811
Cortex	6801 (4022)	8702 (2684)	2.1079	**0.0388**	7499 (3359)	8957 (2663)	1.993	0.0504	7699 (3620)	8817 (2491)	1.5096	0.1359
CSF	5513 (7306)	3823 (3257)	1.3614	0.178	3840 (5287)	4150 (3761)	0.2824	0.7785	3912 (5338)	4099 (3714)	0.1704	0.8652
DGM	846 (614)	867 (384)	0.1785	0.8589	735 (438)	957 (404)	2.1578	**0.0346**	721 (441)	967 (396)	2.4104	**0.0187**
WM	4812 (3394)	4761 (1892)	0.1047	0.9169	4672 (2604)	4810 (1935)	0.2514	0.8023	4627 (2812)	4842 (1718)	0.39	0.6978
Whole Brain with CSF	17527 (3846)	18038 (3469)	0.4382	0.6627	17434 (3108)	18247 (3755)	0.9413	0.35	17325 (3368)	18323 (3577)	1.16	0.2502
Whole Brain without CSF	12014 (7099)	14216 (4015)	1.5774	0.1195	13594 (5575)	14097 (4277)	0.4211	0.675	13412 (6004)	14224 (3832)	0.6805	0.4986

Table S21. Post-Surgical Period Volume Trajectory Differences between Groups with and without Neurodevelopmental Testing.

	Year 1 Attrition				Year 3 Attrition				Year 5 Attrition			
	Without NDT (N=25)	With NDT (N=50)			Without NDT (N=25)	With NDT (N=37)			Without NDT (N=25)	With NDT (N=36)		
	Mean (SD)	Mean (SD)	t-value	p-value	Mean (SD)	Mean (SD)	t-value	p-value	Mean (SD)	Mean (SD)	t-value	p-value
Brainstem	203 (71)	200 (65)	0.1075	0.9151	175 (70)	213 (58)	1.6526	0.1085	193 (50)	206 (73)	0.5702	0.5726
Cerebellum	1387 (701)	1850 (515)	1.9124	0.0651	1467 (605)	1922 (488)	2.3334	**0.0263**	1570 (587)	1901 (523)	1.6913	0.1008
Cortex	6076 (4125)	9713 (2714)	2.6469	**0.0127**	7905 (4097)	9521 (2665)	1.3687	0.1809	7473 (3984)	9964 (2258)	2.2974	**0.0285**
CSF	4316 (6426)	4124 (3414)	0.2258	0.8228	2607 (4442)	4651 (3833)	1.3703	0.1805	2653 (4106)	4825 (3954)	1.5193	0.1388
DGM	1062 (506)	933 (398)	0.7169	0.4788	832 (427)	1013 (395)	1.2115	0.2349	853 (392)	1017 (416)	1.1316	0.2665
WM	3802 (3133)	5881 (2315)	1.85	0.0739	3531 (2741)	6436 (1795)	3.6649	**0.0009**	4219 (2969)	6280 (1855)	2.462	**0.0196**
Whole Brain with CSF	18488 (5384)	20433 (3164)	1.1579	0.2558	17879 (4112)	21093 (2854)	2.6275	**0.0133**	18204 (3695)	21203 (3088)	2.5233	**0.017**
Whole Brain without CSF	14172 (7409)	16308 (3901)	1.0924	0.2831	15272 (6034)	16442 (3934)	0.672	0.5066	15550 (5530)	16377 (4147)	0.4906	0.6272

Table S22. Perioperative Period Volume Trajectory Differences between Groups with and without Neurodevelopmental Testing.

	Year 1 Attrition				Year 3 Attrition				Year 5 Attrition			
	Without NDT (N=25)	With NDT (N=48)			Without NDT (N=25)	With NDT (N=36)			Without NDT (N=25)	With NDT (N=36)		
	Mean (SD)	Mean (SD)	t-value	p-value	Mean (SD)	Mean (SD)	t-value	p-value	Mean (SD)	Mean (SD)	t-value	p-value
Brainstem	166 (541)	93 (567)	0.4292	0.6704	252 (644)	-10 (458)	1.4679	0.1508	34 (529)	160 (578)	0.7007	0.488
Cerebellum	1235 (2041)	-92 (2392)	1.6728	0.103	1198 (2689)	-563 (1862)	2.3877	**0.0223**	865 (2843)	-439 (1725)	1.7292	0.0923
Cortex	7171 (21066)	5447 (33022)	0.0178	0.9859	9139 (33150)	6001 (29748)	0.306	0.7614	14967 (32793)	443 (27984)	1.473	0.1495
CSF	235 (28327)	5867 (30933)	0.3043	0.7627	11450 (34389)	-3443 (27405)	1.4857	0.1461	7660 (32702)	-1522 (29547)	0.9094	0.3692
DGM	-1100 (5115)	608 (3671)	1.2428	0.222	-529 (4770)	882 (3623)	1.0376	0.3064	-80 (4735)	619 (3624)	0.5139	0.6104
WM	481 (27483)	-832 (21705)	0.2314	0.8183	5975 (25724)	-6032 (19698)	1.6309	0.1116	3570 (27921)	-5069 (16936)	1.1667	0.251
Whole Brain with CSF	12408 (18710)	7823 (22258)	0.7389	0.4648	17362 (21389)	1152 (20170)	2.3849	**0.0225**	15616 (21790)	1103 (20225)	2.1293	**0.0401**
Whole Brain without CSF	12173 (24389)	1956 (35529)	0.791	0.4341	5912 (33240)	4595 (32811)	0.1215	0.904	7955 (37343)	2624 (28305)	0.499	0.6208

Table S23. Early Infant Period DTI Trajectory Differences between Groups with and without Neurodevelopmental Testing.

	Year 1 Attrition				Year 3 Attrition				Year 5 Attrition			
	Without NDT (N=25)	With NDT (N=54)			Without NDT (N=38)	With NDT (N=39)			Without NDT (N=37)	With NDT (N=36)		
	Mean (SD)	Mean (SD)	t-value	p-value	Mean (SD)	Mean (SD)	t-value	p-value	Mean (SD)	Mean (SD)	t-value	p-value
FA												
CCBody	0.0022 (0.0009)	0.0024 (0.0011)	0.5592	0.5782	0.0022 (0.0009)	0.0024 (0.0012)	0.893	0.3755	0.0021 (0.0009)	0.0025 (0.0011)	1.559	0.1243
CST-L	0.0044 (0.002)	0.0047 (0.0015)	0.4496	0.6548	0.0045 (0.0016)	0.0047 (0.0015)	0.5091	0.6128	0.0046 (0.0016)	0.0047 (0.0016)	0.0721	0.9428
CST-R	0.0052 (0.0024)	0.0047 (0.0018)	0.7941	0.4307	0.0051 (0.0019)	0.0046 (0.0019)	0.8338	0.4081	0.0051 (0.0018)	0.0046 (0.0019)	0.9716	0.3356
FOF-L	0.0039 (0.0013)	0.003 (0.0011)	2.3172	**0.0242**	0.0037 (0.0012)	0.0029 (0.0011)	2.6478	**0.0105**	0.0036 (0.0012)	0.003 (0.0011)	1.9569	0.0554
FOF-R	0.0034 (0.0017)	0.0031 (0.0012)	0.645	0.522	0.0035 (0.0013)	0.0029 (0.0013)	1.4469	0.1544	0.0033 (0.0013)	0.0031 (0.0013)	0.7313	0.4682
Genu	0.0029 (0.0019)	0.0034 (0.001)	1.2757	0.2072	0.0031 (0.0015)	0.0034 (0.0011)	0.8246	0.413	0.0033 (0.0015)	0.0033 (0.0011)	0.0779	0.9382
ILF-L	0.0043 (0.0009)	0.0038 (0.0013)	1.4117	0.1637	0.0041 (0.001)	0.0037 (0.0013)	1.3262	0.1902	0.0041 (0.001)	0.0038 (0.0014)	0.9129	0.3653
ILF-R	0.0032 (0.0016)	0.0036 (0.0013)	0.843	0.4029	0.0035 (0.0016)	0.0035 (0.0012)	0.1761	0.8609	0.0034 (0.0014)	0.0036 (0.0014)	0.5553	0.581
SLF-L	0.0021 (0.0019)	0.0023 (0.0012)	0.5548	0.582	0.0022 (0.0016)	0.0024 (0.001)	0.5262	0.6015	0.0023 (0.0016)	0.0022 (0.0011)	0.2962	0.7686
SLF-R	0.0026 (0.0016)	0.0029 (0.0016)	0.5804	0.566	0.0027 (0.0013)	0.003 (0.0019)	0.6549	0.5175	0.0025 (0.0014)	0.0032 (0.0018)	1.192	0.2426
Splenium	0.0025 (0.0026)	0.0031 (0.0014)	1.1677	0.2476	0.0032 (0.0018)	0.0028 (0.0017)	0.8432	0.4025	0.0028 (0.0019)	0.0032 (0.0016)	0.9386	0.3518
RD												
CCBody	-0.0076 (0.0077)	-0.0102 (0.0056)	1.3537	0.181	-0.0096 (0.0077)	-0.0097 (0.0046)	0.0945	0.925	-0.0093 (0.0062)	-0.0099 (0.0062)	0.4304	0.6685
CST-L	-0.0109 (0.0071)	-0.0101 (0.0053)	0.4126	0.6816	-0.0105 (0.005)	-0.01 (0.006)	0.3008	0.7648	-0.01 (0.0054)	-0.0104 (0.0058)	0.2758	0.7838
CST-R	-0.0137 (0.0101)	-0.0109 (0.0102)	0.7806	0.4385	-0.0119 (0.0077)	-0.0111 (0.0116)	0.2922	0.7713	-0.0119 (0.008)	-0.0111 (0.0115)	0.2839	0.7776
FOF-L	-0.0108 (0.0045)	-0.0119 (0.0036)	0.8793	0.3831	-0.0113 (0.0042)	-0.0119 (0.0036)	0.5366	0.5937	-0.0115 (0.004)	-0.0118 (0.0037)	0.2443	0.8079
FOF-R	-0.0134 (0.0067)	-0.0132 (0.0034)	0.1806	0.8574	-0.0139 (0.0053)	-0.0127 (0.0032)	1.0036	0.3206	-0.0143 (0.0049)	-0.0124 (0.0034)	1.5989	0.1164
Genu	-0.0128 (0.0038)	-0.0133 (0.0044)	0.3626	0.7182	-0.013 (0.0046)	-0.0133 (0.004)	0.3061	0.7606	-0.0135 (0.0032)	-0.0129 (0.0049)	0.5424	0.5896
ILF-L	-0.0159 (0.0068)	-0.0147 (0.0059)	0.6143	0.5416	-0.0152 (0.006)	-0.0147 (0.0063)	0.2807	0.78	-0.0157 (0.0056)	-0.0143 (0.0065)	0.8465	0.401
ILF-R	-0.0159 (0.0063)	-0.0152 (0.0039)	0.5081	0.6134	-0.0156 (0.0051)	-0.0152 (0.004)	0.34	0.7352	-0.0154 (0.0048)	-0.0153 (0.0043)	0.1029	0.9184
SLF-L	-0.0104 (0.0089)	-0.0106 (0.0047)	0.1314	0.896	-0.0115 (0.0066)	-0.0095 (0.0047)	1.1284	0.2656	-0.0116 (0.0066)	-0.0097 (0.0048)	1.0971	0.2789
SLF-R	-0.0127 (0.0102)	-0.0122 (0.0053)	0.1778	0.86	-0.0121 (0.008)	-0.0126 (0.0055)	0.2343	0.8163	-0.0115 (0.0082)	-0.0133 (0.0049)	0.7476	0.4605
Splenium	-0.0058 (0.0102)	-0.0108 (0.0053)	2.4251	**0.0184**	-0.0096 (0.0088)	-0.0098 (0.0051)	0.0869	0.9311	-0.0084 (0.0078)	-0.0106 (0.0061)	1.2421	0.2191

Table S24. Post-Surgical Period DTI Trajectory Differences between Groups with and without Neurodevelopmental Testing.

	Year 1 Attrition				Year 3 Attrition				Year 5 Attrition			
	Without NDT (N=25)	With NDT (N=50)			Without NDT (N=25)	With NDT (N=37)			Without NDT (N=25)	With NDT (N=36)		
	Mean (SD)	Mean (SD)	t-value		Mean (SD)	Mean (SD)	t-value		Mean (SD)	Mean (SD)	t-value	
FA												
CCBody	0.0029 (0.0014)	0.0028 (0.0011)	0.1099	0.9129	0.0026 (0.0012)	0.003 (0.0011)	1.1665	0.248	0.0027 (0.0012)	0.003 (0.0011)	0.9454	0.3482
CST-L	0.0047 (0.0018)	0.0046 (0.0017)	0.2734	0.7856	0.0047 (0.0017)	0.0045 (0.0017)	0.4918	0.6248	0.0045 (0.002)	0.0046 (0.0015)	0.2122	0.8328
CST-R	0.0062 (0.0024)	0.0051 (0.0021)	1.2282	0.2252	0.0053 (0.0025)	0.0053 (0.0021)	0.0747	0.9408	0.0056 (0.0025)	0.0051 (0.002)	0.7569	0.4527
FOF-L	0.0035 (0.0014)	0.0031 (0.0013)	0.8607	0.3929	0.0035 (0.0015)	0.003 (0.0012)	1.4283	0.1586	0.0034 (0.0014)	0.0031 (0.0013)	0.8301	0.4099
FOF-R	0.0038 (0.0015)	0.0031 (0.0011)	1.5155	0.1356	0.0036 (0.0014)	0.003 (0.001)	1.7059	0.0939	0.0033 (0.0013)	0.0032 (0.001)	0.1773	0.8599
Genu	0.003 (0.0016)	0.0036 (0.0013)	1.321	0.1914	0.0031 (0.0015)	0.0037 (0.0012)	1.574	0.1207	0.0035 (0.0015)	0.0034 (0.0012)	0.0746	0.9408
ILF-L	0.0045 (0.0014)	0.0035 (0.0014)	2.2424	**0.0288**	0.0041 (0.0016)	0.0034 (0.0013)	1.8393	0.071	0.0041 (0.0015)	0.0033 (0.0014)	2.0276	**0.0472**
ILF-R	0.0035 (0.0012)	0.0036 (0.0014)	0.1353	0.8929	0.0035 (0.0015)	0.0037 (0.0014)	0.3473	0.7297	0.0035 (0.0013)	0.0037 (0.0015)	0.423	0.6739
SLF-L	0.0029 (0.002)	0.0025 (0.0015)	0.6285	0.534	0.0024 (0.0018)	0.0028 (0.0014)	0.7169	0.4785	0.0027 (0.0019)	0.0026 (0.0013)	0.1095	0.9135
SLF-R	0.0018 (0.0012)	0.0032 (0.0017)	1.9341	0.0623	0.0026 (0.0017)	0.0031 (0.0017)	0.7175	0.4784	0.0028 (0.0017)	0.003 (0.0017)	0.4129	0.6825
Splenium	0.0029 (0.0024)	0.0033 (0.0013)	0.9023	0.3705	0.0034 (0.0016)	0.0031 (0.0015)	0.7851	0.4354	0.0031 (0.0017)	0.0033 (0.0015)	0.4299	0.6687
RD												
CCBody	-0.0128 (0.0086)	-0.012 (0.0055)	0.4368	0.6638	-0.0122 (0.0072)	-0.0121 (0.0054)	0.0188	0.985	-0.0132 (0.0067)	-0.0113 (0.0056)	1.2119	0.2302
CST-L	-0.0107 (0.0075)	-0.01 (0.0067)	0.2943	0.7697	-0.0104 (0.006)	-0.01 (0.0073)	0.2399	0.8113	-0.0101 (0.0065)	-0.0102 (0.0071)	0.0575	0.9544
CST-R	-0.0145 (0.0085)	-0.0114 (0.0067)	1.1359	0.2615	-0.0121 (0.0068)	-0.0118 (0.0072)	0.1116	0.9116	-0.0123 (0.0064)	-0.0117 (0.0074)	0.3306	0.7423
FOF-L	-0.0132 (0.008)	-0.0117 (0.0043)	0.921	0.3609	-0.0126 (0.0064)	-0.0116 (0.0043)	0.6622	0.5105	-0.0126 (0.0061)	-0.0115 (0.0044)	0.818	0.4167
FOF-R	-0.0131 (0.008)	-0.013 (0.0041)	0.0647	0.9487	-0.0131 (0.0059)	-0.013 (0.0042)	0.1017	0.9194	-0.0132 (0.0056)	-0.0129 (0.0043)	0.1761	0.8609
Genu	-0.0143 (0.0054)	-0.0135 (0.0048)	0.4851	0.6294	-0.0136 (0.0049)	-0.0137 (0.005)	0.1122	0.911	-0.0147 (0.0044)	-0.0128 (0.0052)	1.4987	0.1391
ILF-L	-0.0182 (0.0067)	-0.0146 (0.0064)	1.7331	0.0884	-0.0167 (0.0066)	-0.0144 (0.0064)	1.3847	0.1714	-0.0174 (0.0065)	-0.0136 (0.0062)	2.3629	**0.0215**
ILF-R	-0.0165 (0.0074)	-0.0152 (0.0045)	0.7518	0.4554	-0.0146 (0.006)	-0.016 (0.0044)	1.0244	0.3102	-0.0155 (0.0058)	-0.0154 (0.0046)	0.0477	0.9622
SLF-L	-0.0137 (0.0076)	-0.0122 (0.0056)	0.6624	0.5123	-0.0129 (0.0071)	-0.0123 (0.0052)	0.3088	0.7594	-0.0147 (0.0071)	-0.0106 (0.0042)	2.1053	**0.043**
SLF-R	-0.0114 (0.0108)	-0.0121 (0.0073)	0.213	0.8327	-0.0112 (0.0108)	-0.0126 (0.0049)	0.5109	0.613	-0.013 (0.0075)	-0.0107 (0.0087)	0.8159	0.4208
Splenium	-0.0074 (0.0088)	-0.0109 (0.0046)	2.0055	**0.0494**	-0.0104 (0.0067)	-0.0101 (0.0052)	0.187	0.8523	-0.0108 (0.0064)	-0.0097 (0.0053)	0.7013	0.4858

Table S25. Perioperative Period DTI Trajectory Differences between Groups with and without Neurodevelopmental Testing.

	Year 1 Attrition				Year 3 Attrition				Year 5 Attrition			
	Without NDT (N=25)	With NDT (N=48)			Without NDT (N=25)	With NDT (N=36)			Without NDT (N=25)	With NDT (N=36)		
	Mean (SD)	Mean (SD)	t-value	p-value	Mean (SD)	Mean (SD)	t-value	p-value	Mean (SD)	Mean (SD)	t-value	p-value
FA												
CCBody	-0.0003 (0.0173)	-0.0033 (0.0169)	0.7008	0.4857	-0.0016 (0.019)	-0.003 (0.0147)	0.3404	0.7346	-0.0019 (0.018)	-0.0027 (0.0162)	0.1955	0.8456
CST-L	0.0049 (0.0153)	0.0035 (0.0231)	0.2553	0.7993	0.0049 (0.0218)	0.003 (0.02)	0.3763	0.7079	0.0051 (0.0206)	0.0027 (0.0212)	0.4815	0.6317
CST-R	0.0009 (0.0196)	-0.0012 (0.0246)	0.3094	0.7581	0.0034 (0.0222)	-0.0036 (0.0236)	1.1608	0.2505	0.0079 (0.0207)	-0.0066 (0.023)	2.5192	**0.0145**
FOF-L	0.0051 (0.0083)	0.0032 (0.0176)	0.4743	0.6368	0.0041 (0.0195)	0.0036 (0.0095)	0.1549	0.8774	0.0061 (0.0186)	0.0015 (0.0102)	1.2298	0.2232
FOF-R	0.0018 (0.0085)	0.0028 (0.0161)	0.2822	0.7788	0.0031 (0.0181)	0.0018 (0.0081)	0.3849	0.7017	0.004 (0.0175)	0.0007 (0.008)	0.9137	0.3645
Genu	0.0033 (0.0152)	0.0007 (0.0175)	0.6196	0.5375	0.0051 (0.0185)	-0.0019 (0.0139)	1.819	0.0731	0.0021 (0.0169)	0.0011 (0.0166)	0.2367	0.8135
ILF-L	0.0036 (0.0101)	0.0061 (0.0165)	0.6729	0.5034	0.0051 (0.017)	0.0054 (0.0119)	0.0913	0.9276	0.0045 (0.0171)	0.006 (0.0118)	0.4384	0.6625
ILF-R	-0.0012 (0.0106)	0.0037 (0.015)	1.3428	0.1842	0.0031 (0.0177)	0.0011 (0.0085)	0.5882	0.5585	0.0023 (0.0169)	0.0019 (0.01)	0.0982	0.9221
SLF-L	-0.0031 (0.0162)	0.0018 (0.0287)	0.5698	0.5723	0.0052 (0.0268)	-0.0063 (0.0216)	1.4287	0.1617	0.0037 (0.0278)	-0.0039 (0.0215)	0.9337	0.3567
SLF-R	-0.0049 (0.0204)	0.0011 (0.0233)	0.5701	0.5742	-0.0065 (0.0244)	0.0064 (0.0188)	1.4697	0.1552	-0.006 (0.0218)	0.0118 (0.0197)	1.9606	0.0621
Splenium	-0.0014 (0.0142)	0.0003 (0.0175)	0.4354	0.6646	-0.0012 (0.0132)	0.0007 (0.0193)	0.4854	0.6289	-0.0008 (0.0154)	0.0002 (0.0175)	0.2489	0.8041
RD												
CCBody	0.0186 (0.0815)	0.0183 (0.086)	0.0137	0.9891	0.016 (0.1117)	0.0209 (0.0386)	0.2534	0.8007	0.0271 (0.0732)	0.0097 (0.0936)	0.8885	0.3772
CST-L	-0.0101 (0.0768)	0.0021 (0.1104)	0.4648	0.6436	-0.0145 (0.0533)	0.01 (0.1299)	1.0057	0.3182	-0.0004 (0.0634)	-0.0034 (0.1296)	0.1228	0.9026
CST-R	0.0147 (0.1977)	0.0096 (0.1345)	0.1163	0.9078	-0.0121 (0.1567)	0.0289 (0.1525)	1.0191	0.3124	-0.0096 (0.1491)	0.026 (0.1585)	0.8808	0.382
FOF-L	0.0074 (0.0735)	-0.0159 (0.0494)	1.5364	0.1293	0.0006 (0.0698)	-0.0168 (0.0453)	1.2147	0.2289	-0.0021 (0.0693)	-0.0145 (0.046)	0.8658	0.3898
FOF-R	-0.0123 (0.0336)	-0.0139 (0.0496)	0.1397	0.8894	-0.0178 (0.048)	-0.009 (0.041)	0.775	0.4414	-0.0175 (0.0467)	-0.0086 (0.0422)	0.7833	0.4365
Genu	-0.0089 (0.0404)	-0.0025 (0.0402)	0.639	0.5249	-0.0093 (0.0462)	0.0001 (0.0327)	1.0038	0.3189	-0.005 (0.0457)	-0.0043 (0.034)	0.0741	0.9412
ILF-L	0.0141 (0.0833)	0.0025 (0.0721)	0.5995	0.5509	0.0143 (0.0912)	-0.0015 (0.0562)	0.856	0.3951	0.0026 (0.0821)	0.0102 (0.0695)	0.4129	0.681
ILF-R	-0.0063 (0.0552)	-0.0133 (0.0595)	0.4566	0.6495	-0.0199 (0.0729)	-0.0021 (0.0361)	1.2333	0.2221	-0.0156 (0.0622)	-0.0064 (0.0536)	0.63	0.531
SLF-L	0.0166 (0.1098)	0.0168 (0.0825)	0.0081	0.9936	-0.008 (0.0952)	0.0473 (0.0783)	1.9229	0.0624	-0.0041 (0.0951)	0.0399 (0.0832)	1.5127	0.1391
SLF-R	-0.0417 (0.102)	0.0132 (0.095)	1.2143	0.2369	0.0009 (0.1209)	-0.0009 (0.0693)	0.045	0.9645	0.0132 (0.1087)	-0.0279 (0.0654)	0.9836	0.3356
Splenium	-0.0005 (0.0564)	0.002 (0.0944)	0.1179	0.9064	-0.0043 (0.1067)	0.0069 (0.0478)	0.5738	0.5679	0.0125 (0.0619)	-0.0102 (0.0995)	1.1815	0.2413

Table S26. Sociodemographic Factor Differences between Groups with and without Neurodevelopmental Testing.

		With NDT	Without NDT
	p-value	mean (SD)	mean (SD)
5-Year		N=36	N=37
SES	0.3654	41.09 (13.50)	37.00 (16.65)
Maternal IQ	0.5233	104.08 (17.92)	101.05 (15.10)
Latino	0.4718	8	10
White	0.2567	26	22
Black	0.2544	2	5
Asian – only 2 were recruited but did not attend ND testing			
3-Year		N=39	N=38
SES	0.0941	41.89 (13.78)	34.08 (15.35)
Maternal IQ	0.2198	104.90 (16.87)	98.94 (16.75)
Latino	0.3974	8	11
White	0.1300	29	22
Black	0.2258	2	5
Asian – only 2 were recruited but did not attend ND testing			
1-Year		N=54	N=25
SES	0.5047	39.51 (14.48)	45.33 (16.04)
Maternal IQ	0.3315	102.5 (17.01)	112.33 (14.36)
Latino	0.8808	15	6
White	0.2265	32	17
Black	0.9383	5	2
Asian – only 2 were recruited but did not attend ND testing			

Table S27. One-year and Three-year Neurodevelopmental differences between Participants that Attended Five-year Assessment and Those Who Did Not.

		Had 5-Year NDT	Without 5-Year NDT
	p-value	mean (sd)	mean (sd)
3-Year		N=32	N=5
Bayley-III Language	0.8242	96.19 (13.66)	97.6 (7.77)
Bayley-III Cognitive	0.3654	99.22 (10.56)	94.6 (9.79)
Bayley-III Motor	0.5634	100.66 (12.91)	97.2 (6.02)
1-Year		N=36	N=18
Bayley-III Language	0.6483	89.73 (11.97)	91.7 (12.31)
Bayley-III Cognitive	0.0568	104.59 (12.93)	96.36 (9.51)
Bayley-III Motor	0.1184	92.7 (12.75)	85.6 (11.56)

Supplemental References

1. Ceschin R, Zahner A, Reynolds W, et al. A computational framework for the detection of subcortical brain dysmaturation in neonatal MRI using 3D Convolutional Neural Networks. *NeuroImage*. 2018;178:183-197.

2. Jenkinson M, Bannister P, Brady M, Smith S. Improved optimization for the robust and accurate linear registration and motion correction of brain images. *Neuroimage*. 2002;17(2):825-841.

3. Zhang Y, Brady M, Smith S. Segmentation of brain MR images through a hidden Markov random field model and the expectation-maximization algorithm. *IEEE transactions on medical imaging*. 2001;20(1):45-57.

4. Gousias IS, Hammers A, Counsell SJ, et al. Magnetic resonance imaging of the newborn brain: automatic segmentation of brain images into 50 anatomical regions. *PloS one*. 2013;8(4):e59990.

5. Serag A, Aljabar P, Ball G, et al. Construction of a consistent high-definition spatio-temporal atlas of the developing brain using adaptive kernel regression. *Neuroimage*. 2012;59(3):2255-2265.

6. Avants B, Duda JT, Kim J, et al. Multivariate analysis of structural and diffusion imaging in traumatic brain injury. *Academic radiology*. 2008;15(11):1360-1375.

7. Yeh F-C, Verstynen TD, Wang Y, Fernández-Miranda JC, Tseng W-YI. Deterministic diffusion fiber tracking improved by quantitative anisotropy. *PloS one*. 2013;8(11):e80713.

8. Woolrich MW, Jbabdi S, Patenaude B, et al. Bayesian analysis of neuroimaging data in FSL. *Neuroimage*. 2009;45(1):S173-S186.

9. Andersson JL, Jenkinson M, Smith S. Non-linear registration, aka Spatial normalisation FMRIB technical report TR07JA2. *FMRIB Analysis Group of the University of Oxford*. 2007;2(1):e21.

10. Avants BB, Tustison NJ, Song G, Cook PA, Klein A, Gee JC. A reproducible evaluation of ANTs similarity metric performance in brain image registration. *Neuroimage*. 2011;54(3):2033-2044.

11. McCarthy AL, Winters ME, Busch DR, et al. Scoring system for periventricular leukomalacia in infants with congenital heart disease. *Pediatric research*. 2015;78(3):304-309.
